# Modulating the Photocyclization Reactivity of Diarylethenes through Changes in the Excited-State Aromaticity of the π-Linker

**DOI:** 10.1021/acs.joc.2c01172

**Published:** 2022-08-23

**Authors:** Baswanth Oruganti, Jun Wang, Bo Durbeej

**Affiliations:** † Division of Theoretical Chemistry, IFM, Linköping University, Linköping SE-58183, Sweden; ‡ Department of Chemistry, SRM University-AP, Mangalagiri, Andhra Pradesh 522240, India; § Jiangsu Key Laboratory for Chemistry of Low-Dimensional Materials, Jiangsu Engineering Laboratory for Environment Functional Materials, School of Chemistry and Chemical Engineering, Huaiyin Normal University, Huaian 223300, China

## Abstract

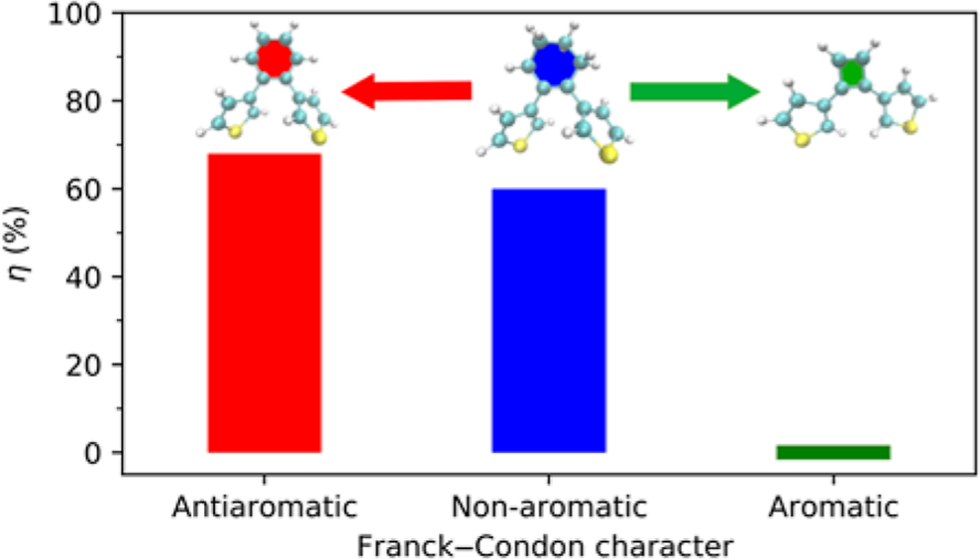

Quantum chemical calculations are performed to explore if the reactivity of diarylethene switches toward photocyclization can be controlled by the excited-state aromaticity of their bridging π-linker. Using an archetypal diarylethene with a non-aromatic π-linker as a reference, completely different outcomes are found when the π-linker is allowed to become either aromatic (no reaction) or antiaromatic (fast reaction) upon photoexcitation. The results demonstrate a possibility to use the excited-state aromaticity concept for actual modulation of photochemical reactivity.

## Introduction

The use of the excited-state aromaticity (ESA) and antiaromaticity (ESAA) concepts^1^ to describe cyclic, conjugated organic molecules in their electronically excited states has in the past decade or so witnessed a resurgence,^2^ helping explain and predict both photophysical properties and photochemical reactivity of such compounds in a variety of contexts. For example, as for photophysical properties, these concepts have been employed to explain differences in Stokes shifts of benzoxazole fluorophores,^3^ to design chromophores with singlet-triplet energy gaps amenable to singlet-fission photovoltaics,^4^ and to design red-light fluorophores based on a simple benzene core.^5^ Regarding photochemical reactivity, in turn, the concepts have proven very useful in finding ways to improve the quantum yields of *E*/*Z* photoisomerizations that power light-driven molecular motors,^6^ in identifying the driving force for excited-state proton transfer^7^ and conformational planarization^8^ reactions, and in understanding the mechanisms of electron-catalyzed photodissociation processes.^9^

As for the impact of aromaticity on other types of photochemical reactions, which has been less investigated, electrocyclic reactions of molecular photoswitches are a curious case in that the presence of an aromatic moiety can have both positive^10^ and negative^11^ consequences for these transformations. As an example of the former situation, a study of diarylethene switches^12^ recently presented both experimental and computational evidence indicating that the insertion of benzene as the bridging π-linker between the two aryl units (in the form of thienyl groups) facilitates the photocyclization of the ring-open form of the corresponding dithienylbenzene into its ring-closed form.^10^ Specifically, it was found that the photocyclization is driven by the complete loss of aromaticity in the benzene moiety upon photoexcitation, which creates a reactive, antiaromatic excited state in the Franck–Condon (FC) region that allows ring-closing to occur in an energetically downhill fashion through the subsequent relief of this antiaromaticity.^10^ Contrarily, in a study of the photoinduced conversion of a benzannulated dihydroazulene switch into the corresponding vinylheptafulvene isomer, it was noted that the loss of aromaticity in the benzene motif from the initial electronic excitation is only partial, whereby the relief of the remaining aromaticity during the subsequent excited-state evolution introduces a barrier for the reaction.^11^

**Scheme 1:**
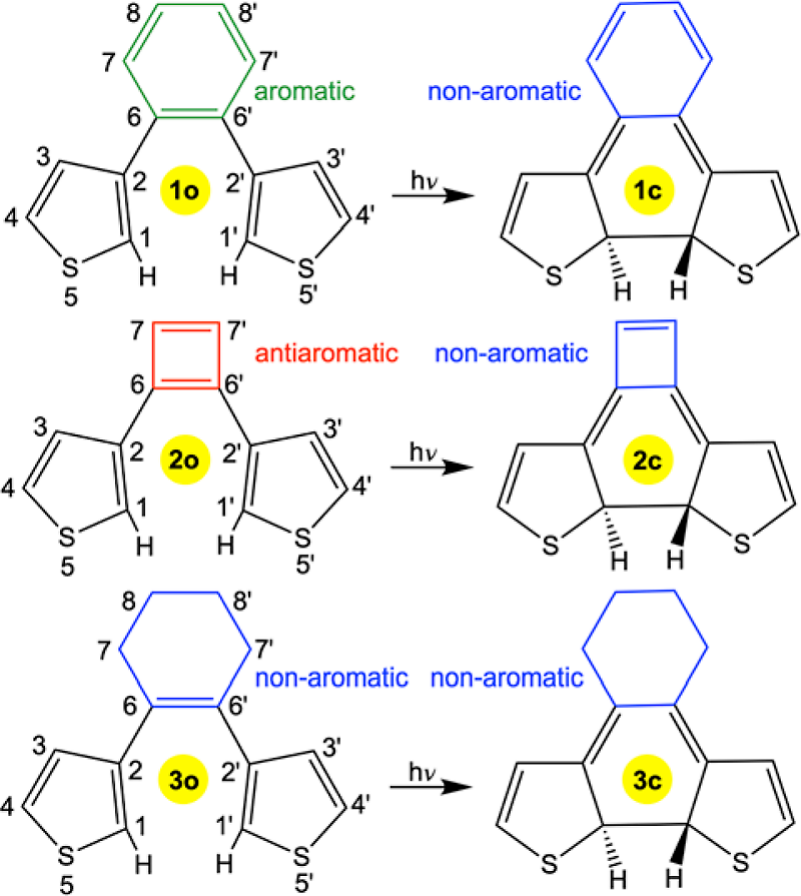
Photocyclization Reactions Studied in This Work

In light of these findings that photoinduced changes in aromaticity can influence electrocyclic reactions of different photoswitches so differently, it becomes pertinent to explore the extent to which it is possible to control these reactions by modulating the ESA among a specific type of switches. The present work reports the first investigation of this important but hitherto unresolved problem. To this end, we here model the photocyclization reactions of the three diarylethenes shown in their ring-open (**1o**/**2o**/**3o**) and ring-closed (**1c**/**2c**/**3c**) isomeric forms in Scheme 1, by performing both quantum chemical calculations and non-adiabatic molecular dynamics (NAMD) simulations.^13^**1o** is the aforementioned dithienylbenzene switch featuring an aromatic benzene bridge that becomes antiaromatic in the FC region of the lowest singlet excited state (S_1_) populated by UV absorption.^10^**2o**, in turn, is a dithienylcyclobutadiene compound where the benzene bridge is replaced by an antiaromatic cyclobutadiene motif that, conversely, turns aromatic upon UV absorption (this will be demonstrated below). **3o**, finally, is a dithienylcyclohexene compound where benzene instead is replaced by a non-aromatic cyclohexene motif that remains non-aromatic after UV absorption (this will also be demonstrated below).

With these systems, our work encompasses all three relevant scenarios for the character of the photoactive excited state in the FC region, ranging from antiaromatic in **1o** to non-aromatic in **3o** and aromatic in **2o**. By comparing the results for **1o** and **2o**, the calculations will reveal whether the photocyclization reactivity is indeed tunable by ESA. Furthermore, by comparing the results for **1o**/**2o** with those for **3o**, whose non-aromatic cyclohexene bridge best represents available diarylethene switches^12^ (including systems with a perfluorocyclohexene bridge^14^) considered for applications in, for example, molecular electronics^15^ and photopharmacology,^16^ the calculations will also assess whether the photocyclization reactivity achieved by ESA-tuned diarylethenes parallels that achieved by typical diarylethenes.

## Results and Discussion

The full technical details of the modeling, carried out at levels of theory previously benchmarked against experimental UV–vis absorption data,^10^ are given in the Supporting Information.

### Static Modeling of Photocyclization Reactions

**Figure 1:**
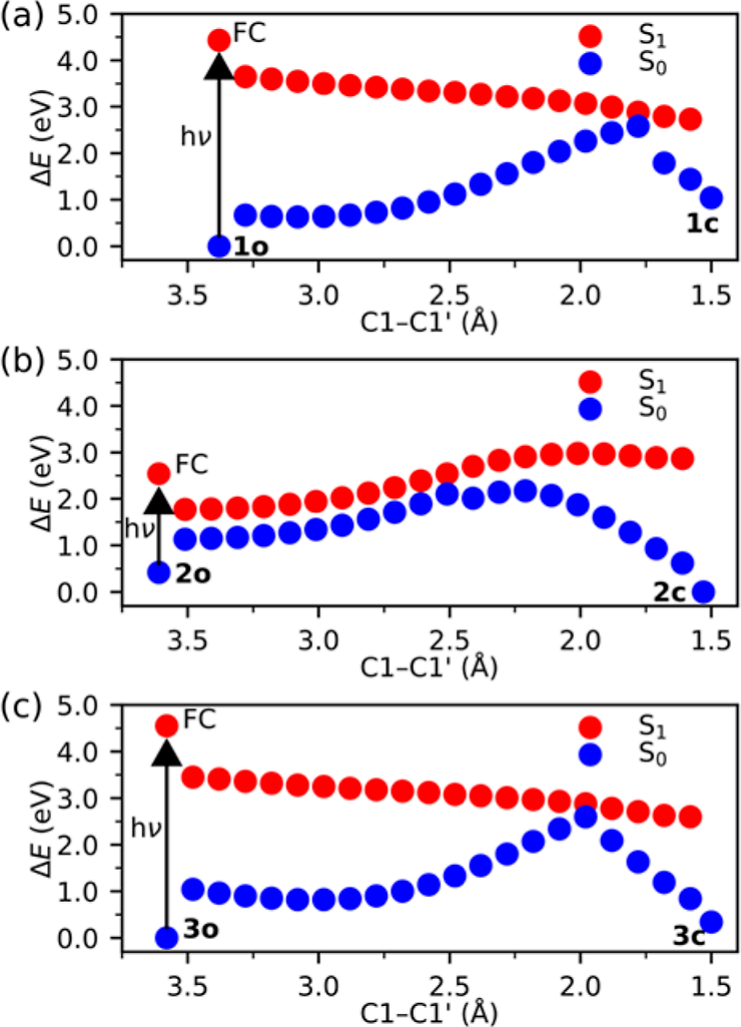
Calculated photocyclization paths in the S_1_ state of **1o** (a), **2o** (b), and **3o** (c) with energies (Δ*E*) in each case given relative to the most stable species in the S_0_ state (**1o**, **2c**, and **3o**, respectively). Also shown are the S_0_ energies at the S_1_ geometries along the photocyclization paths. Complementary calculations performed using other (than B3LYP) quantum chemical methods that are discussed in the Supporting Information (see Tables S2 and S3) corroborate the shapes of these paths.

First, the photocyclization reactions of **1o**/**2o**/**3o** into **1c**/**2c**/**3c** were modeled by performing quantum chemical calculations using the B3LYP hybrid density functional in combination with the cc-pVTZ basis set and the SMD continuum solvation approach^17^ to model an acetonitrile solvent, which was used in the aforementioned experimental studies of **1o**.^10^ In these calculations, which are summarized in Figure 1, the S_1_ state of the different species was described in the framework of time-dependent density functional theory (TD-DFT).^18^ Besides modeling the photocyclization processes, the reaction free energies and free energy barriers for the corresponding thermal electrocyclization and cycloreversion (i.e., ring-opening) processes in the ground S_0_ state were also calculated (at the same level of theory). These results are presented in Figure S1 and Table S1 in the Supporting Information.

From Figure 1, wherein the reaction coordinate starting from the vertically excited S_1_ FC point is taken to be the C1–C1′ distance (the atom numbering is given in Scheme 1), it can first be seen that the calculated photocyclization paths of **1o** and **2o** are distinctly different. For **1o**, the reaction is completely barrierless and enables the S_1_ state to gradually come closer and closer to the S_0_ state in a region of a presumed S_1_/S_0_ conical intersection (CI) seam that connects the photocyclization path to **1c**. Notably, reinforcing our previous results on **1o** obtained using a different density functional than B3LYP (ωB97X-D),^10^ the motion toward this seam is found to have an appreciable driving force, as revealed by the fact that the S_1_ energy at C1–C1′ = 1.78 Å (where the S_1_/S_0_ gap is the smallest) lies 1.55 eV below the FC point at 3.38 Å. For **2o**, on the other hand, there is a minimum at 3.55 Å along the photocyclization path, in the near vicinity of the FC point at 3.61 Å, and any further motion toward shorter C1–C1′ distances is impeded by a continuous increase in the S_1_ energy. Accordingly, from this comparison of **1o** and **2o**, it does appear that the photocyclization reactivity of diarylethene switches can be tuned through the aromatic character of the π-linker between the two aryl units. Moreover, since the calculated photocyclization path of **1o** has the same favorable features as that of **3o** (see Figure 1), which represents a typical diarylethene with a non-aromatic π-linker,^12^ it seems possible for diarylethenes that are tuned in this way to be fully competitive in terms of their intrinsic photochemical performance.

Regarding the thermal electrocyclization reactions, in turn, the calculated free energies are markedly different for **1o** and **2o** (see Table S1), with **1o** having a very large barrier (181 kJ mol^–1^) and a distinctly positive reaction energy (112 kJ mol^–1^), and **2o** showing a much smaller barrier (104 kJ mol^–1^) and a negative reaction energy (−24 kJ mol^–1^). This difference reflects that the π-linker loses aromaticity in the reaction of **1o** and antiaromaticity in the reaction of **2o**. Furthermore, it is also of interest to compare the +112 kJ mol^–1^ reaction energy of **1o** with the +46 kJ mol^–1^ reaction energy of **3o**, which has a non-aromatic π-linker. This comparison reveals that diarylethenes such as **1o**/**1c** are an interesting prospect for applications in molecular solar thermal energy storage,^19^ because of the high energy content of their ring-closed forms. At the same time, such applications would require the ring-closed forms to be stable toward thermal cycloreversion, which could be a challenge. Indeed, the calculations predict that **1c** is more susceptible to this process than **3c** (cf. barriers of 70 and 142 kJ mol^–1^, respectively, in Table S1). A similar trade-off has previously been documented for related diarylethenes.^20^

### NAMD-Based Modeling of Photocyclization Reactions

Although Figure 1 offers a clear picture of the differences between **1o** and **2o** and of the similarities between **1o** and **3o** as to their photocyclization reactivity, it should be noted that the underlying calculations are based on a predefined C1–C1′ reaction coordinate, whereby any influence of competing side reactions is neglected. Moreover, because of their static nature, calculations of this type cannot predict the time scales over which the photocyclizations occur. Therefore, in order to circumvent these limitations, NAMD simulations of **1o**–**3o** were performed for maximally 300 fs at the B3LYP/cc-pVDZ level of theory using Tully’s fewest switches algorithm,^21^ as further described in the Supporting Information. These simulations extend the scope of our previous NAMD-based study of the photocyclization dynamics of **1o**,^10^ both by now considering diarylethenes with different excited-state character and by running many more trajectories (25 instead of 10), whereby the reaction efficiency can be assessed with a higher degree of statistical certainty.

**Figure 2:**
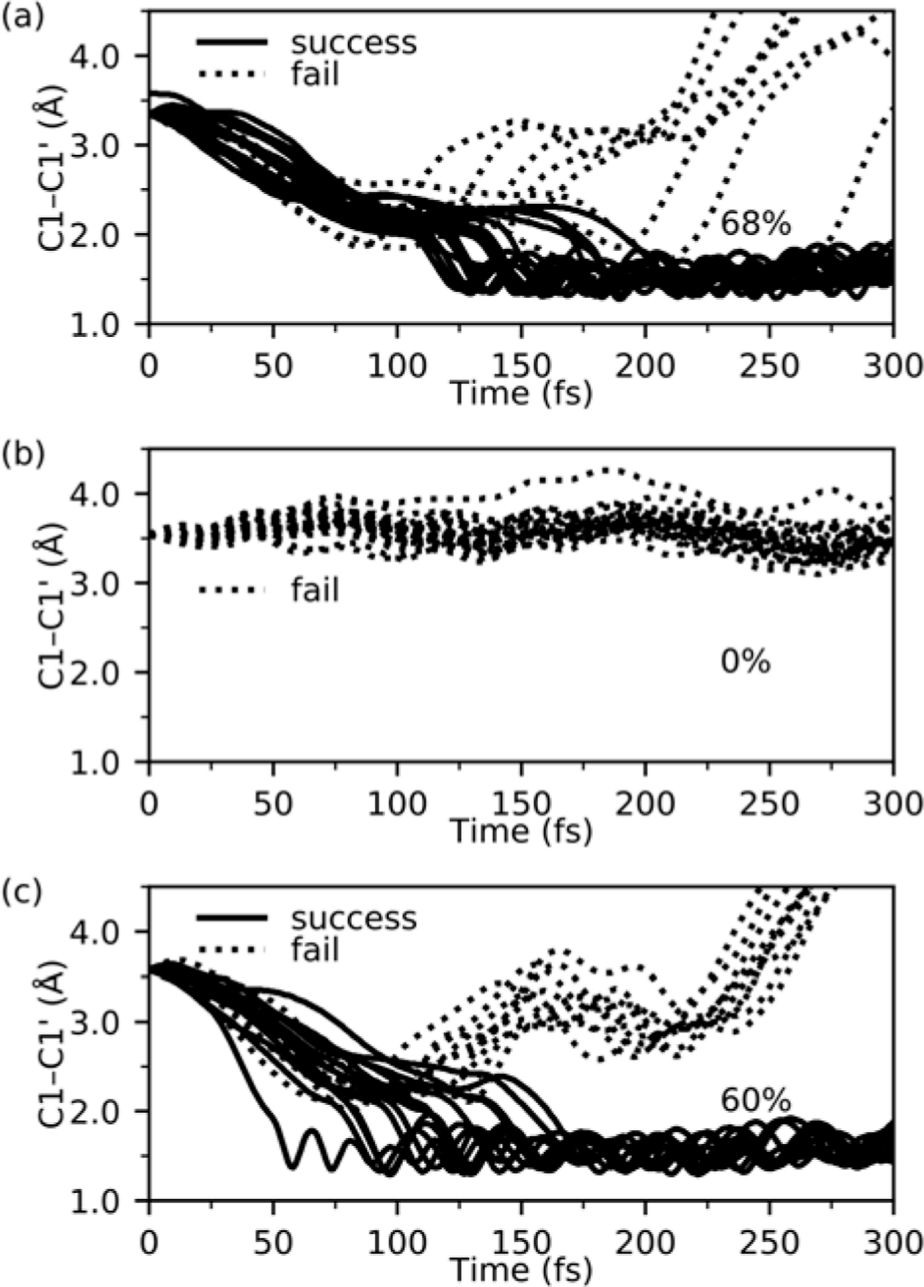
Changes in C1–C1′ distances during the 25 NAMD trajectories run for each of **1o** (a), **2o** (b), and **3o** (c). As described in the NAMD section of the Supporting Information, the trajectories are separated into successful and failed ones that, respectively, complete and do not complete photocyclization within 300 fs. Percentage values refer to the proportions of successful trajectories.

The results from the NAMD simulations, launched at the respective S_1_ FC point, are summarized in Figure 2 and Figures S3 and S4 in the Supporting Information. Starting with the **2o** system, Figure S3b shows that the S_1_ and S_0_ states approach degeneracy almost instantly in the simulations, in keeping with the small magnitudes of the S_1_/S_0_ energy gaps along the corresponding photocyclization path in Figure 1b. However, as revealed by Figure 2b, none of the **2o** trajectories evolve toward shorter C1–C1′ distances, which is consistent with the photocyclization path being energetically unfavorable. Combined with the fact that all 25 trajectories decay to the S_0_ state already within 25 fs (see Figure S4b), this means that there is no dynamical preference for ring-closing to occur. Thus, rather than producing the **2c** photoproduct, the excited-state dynamics reforms the initial **2o** species.

Continuing with the **1o** and **3o** systems, the situation is markedly different. Here, the fast (<100 fs) approaching of degeneracy observed in Figure S3a,c is accompanied by pronounced decreases in the C1–C1′ distances (see Figure 2a,c), which conform to the barrierless shapes of the corresponding photocyclization paths (see Figure 1a,c) and show that, for both systems, ring-closing is a major component of the excited-state dynamics. In fact, after 200 fs, almost all of the two sets of trajectories reside in the S_0_ state (see Figure S4a,c) and more than half oscillate around a C1–C1′ distance of 1.5 Å (see Figure 2a,c). In other words, within 200 fs, more than half of the trajectories complete the photocyclizations by producing the **1c** and **3c** photoproducts, seemingly without any strong competition from side reactions. Qualitatively, this time scale agrees with experimental measurements of the ring-closing dynamics of diarylethenes switches in different solvents.^22^ At the end of the simulations, 68 and 60% of the trajectories have formed the **1c** and **3c** photoproducts, respectively, with the two hydrogen atoms at the C1 and C1′ carbons consistently adopting an *anti* conformation (see Scheme 1) as a result of the ring-closing occurring in a conrotatory fashion, in accordance with the Woodward–Hoffmann rules. Although the slightly lower efficiency of the reaction of **3o** is compensated for by it being somewhat faster (the average photocyclization time, calculated as described in the NAMD section of the Supporting Information, is ∼30 fs shorter for **3o** than for **1o**), these differences between the two systems are too small to allow for any quantitative investigation of their origin by the present simulations.

### Calculated Aromaticity Indices

Having found by both static quantum chemical calculations and NAMD simulations that **1o** and **2o** are very different with respect to their photocyclization reactivity, it remains to establish whether this can be understood in terms of a difference in the aromatic character of their π-linkers following photoexcitation. In fact, such a difference can be anticipated from the observation in Figure 1 that the photon energy required to excite **2o** is only half of that needed to excite **1o**. Accordingly, for **2o**, the photon energy may be insufficient to overcome any barrier for the photocyclization.

**Figure 3:**
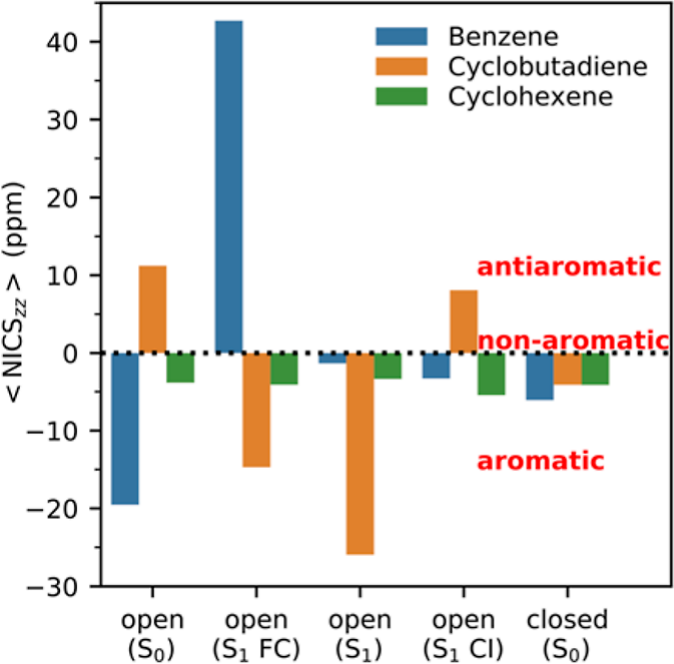
<NICS_zz_> values for the benzene/cyclobutadiene/cyclohexene π-linkers of **1o**/**2o**/**3o** calculated at key points along the corresponding photocyclization paths. The points are presented from left to right in the order in which they appear along the paths (see Table S4 for the corresponding C1–C1′ distances).

To this end, the complete active space self-consistent field (CASSCF) method^23^ was used to calculate nucleus-independent chemical shift (NICS) indices^24^ for the π-linkers of **1o** and **2o** (as well as **3o**) at key points along the corresponding photocyclization paths, as further detailed in the Supporting Information. Briefly, providing a magnetic measure of aromaticity, these indices were calculated through a NICS-scan procedure^25^ to obtain NICS_zz_ values^24b^ at distances 1.50/1.60/1.70/1.80/1.90/2.00 Å above the geometric centers of the π-linkers. This procedure was applied equally to the S_0_ and S_1_ states and was adopted to avoid having to choose (more arbitrarily) one specific distance at which to calculate a single NICS_zz_ value.^25^ Furthermore, the rationale for choosing the current scan interval is to simultaneously minimize both the contributions from σ-electrons to the induced magnetic field^25b^ and the offset between the center of the field and the normal axis passing through the geometric center of the π-linker.^25d,25e^ For ease of analysis, the resulting values were averaged over the different distances to yield mean NICS_zz_ values (<NICS_zz_>) that are presented in Figure 3. The full set of NICS_zz_ values are given in Table S4 in the Supporting Information. Moreover, the Supporting Information also includes a discussion of results (summarized in Figures S5 and S6) from calculations of electronic aromaticity indices that support the NICS-based analysis below.

Noting that negative/positive NICS values reflect aromaticity/antiaromaticity,^24a^Figure 3 clearly indicates that the difference in photocyclization reactivity between **1o** and **2o** can be explained from the aromatic character of their π-linkers following photoexcitation. Specifically, while photoexcitation of **1o** generates a reactive excited state through the conversion of its benzene π-linker into an antiaromatic moiety in the S_1_ FC region (the <NICS_zz_> value changes from −20 to 43 ppm), photoexcitation of **2o** conversely produces a non-reactive excited state via the transformation of its cyclobutadiene π-linker into an aromatic motif in the S_1_ FC region (the <NICS_zz_> value changes from 11 to −15 ppm). At the nearby minimum along the photocyclization path of **2o** (see Figure 1b), this aromaticity is further strengthened (the <NICS_zz_> value is lowered to −26 ppm), which explains why any additional ring-closing motion beyond the minimum is energetically unfavorable.

In sharp contrast to the distinct photoinduced changes in aromaticity shown by **1o** and **2o**, the cyclohexene π-linker of **3o** maintains its non-aromatic character throughout the photocyclization process, consistently exhibiting <NICS_zz_> values close to zero (see Figure 3). Pleasingly, the stark differences among **1o**–**3o** documented in Figure 3 are further reinforced by complementary NICS_zz_ calculations, as summarized in Figure S7 in the Supporting Information, in which the aforementioned 1.50–2.00 Å scan interval was extended to 1.00–2.50 Å. In this regard, it may be noted that it is not uncommon to consider even shorter distances than 1.00 Å for such calculations.^25c^ However, as alluded to above, this will typically exaggerate predictions of aromatic/antiaromatic character, because of the ensuing contamination of the induced magnetic field by σ-electrons.^25b^ At the same time, at short distances, this effect is somewhat countered by the offset between the center of the field and the geometric ring center, which tends to lead to an underestimation of the strength of the field.^25d,25e^

Besides photoinduced aromaticity, another potential reason for the non-reactivity of **2o** is ring strain in the four-membered π-linker. In order to assess this possibility, a photocyclization path was also calculated for a diarylethene switch **4o** in which the antiaromatic cyclobutadiene π-linker of **2o** is replaced by another four-membered motif (cyclobutene, see Figure S8a in the Supporting Information) that is non-aromatic and remains non-aromatic during the reaction. The resulting path is shown in Figure S8b in the Supporting Information. Notably, the path is completely barrierless. Hence, it seems unlikely that ring strain is a reason for the non-reactivity of **2o**. Rather, the only discernible impediment to the photocyclization of **2o** is the photoinduced aromaticity of the cyclobutadiene π-linker.

As indicated above and discussed in the Supporting Information, the scenarios predicted by the NICS data in Figure 3 are corroborated by the calculation of electronic aromaticity indices. Particularly, focusing on Shannon aromaticity (SA) indices,^26^ the −log_10_(SA) values for the different π-linkers in Figure S6 confirm that the photoexcited S_1_ state of **1o**/**2o** experiences a marked loss/gain in aromaticity relative to the S_0_ state (the values decrease/increase by more than 1 unit), whereas the character of the S_1_ state of **3o** is altered to a lesser extent, seemingly becoming somewhat more aromatic (the values increase by ∼0.5 units). At the same time, even these relatively minor increases in the −log_10_(SA) values may be too large to support quantitatively the prediction by the NICS data that the cyclohexene π-linker of **3o** remains, in an absolute sense, non-aromatic in S_1_. In this regard, it is worth noting that, especially for excited states, there are no well-established boundaries between aromatic/non-aromatic/antiaromatic systems in terms of their typical −log_10_(SA) values.

## Conclusions

In summary, based on quantum chemical calculations and NAMD simulations, we have discovered that it is possible to tune the photocyclization reactivity of diarylethene switches by modulating the ESA of the π-linker between the two aryl units. Furthermore, we have found that this tuning can be implemented without taking away from the reactivity achieved by archetypal diarylethenes^12^ featuring a non-aromatic π-linker. Given the many possible areas of application for these switches,^12,15,16^ a natural goal of future research is to also find ways to maximize their reactivity through this approach.

## Computational Methods

The full computational details are given in the Supporting Information. Briefly, the photocyclization reactions of **1o**/**2o**/**3o** into **1c**/**2c**/**3c** were modeled with DFT and TD-DFT^18^ by performing static quantum chemical calculations and NAMD simulations at the B3LYP/cc-pVTZ/SMD and B3LYP/cc-pVDZ levels of theory, respectively. NICS indices at key points along the respective photocyclization path were calculated using CASSCF/cc-pVDZ and (12,12)/(12,12)/(10,10) active spaces for the reactions of **1o**/**2o**/**3o**. The corresponding SA indices, in turn, were calculated at the B3LYP/cc-pVTZ/SMD level of theory. Static DFT and TD-DFT calculations were performed using Gaussian 16.^27^ NAMD simulations were performed using TURBOMOLE 7.4.^28^ NICS indices were calculated using Dalton 2016.2.^29^ SA indices were calculated using Multiwfn 3.7.^30^

## Supporting Information Available

Computational details, Cartesian coordinates and electronic energies of different geometries of **1**–**3**, and additional results (stationary points and free energies for thermal electrocyclization and cycloreversion reactions, vertical excitation energies and FC relaxation energies, molecular orbitals, analyses of NAMD trajectories, aromaticity indices, and photocyclization path of **4o**) (PDF)
Animation of a representative trajectory from the NAMD simulations of **1o** (MP4)
Animation of a representative trajectory from the NAMD simulations of **2o** (MP4)
Animation of a representative trajectory from the NAMD simulations of **3o** (MP4)
