# Supplementary material for: Modulating the Photocyclization Reactivity of Diarylethenes through Changes in the Excited-State Aromaticity of the π-Linker
Source: J Org Chem. 2022 Aug 23;87(17):11565–71. doi: 10.1021/acs.joc.2c01172 (PMC9442643; doi:10.1021/acs.joc.2c01172)
Supplement: Supplementary file 1 — jo2c01172_si_001.pdf [file jo2c01172_si_001.pdf]

Modulating the Photocyclization Reactivity of Diarylethenes through Changes in the Excited-State Aromaticity of the π-Linker

Baswanth Oruganti,*,†,‡ Jun Wang,§ and Bo Durbeej*,†

†Division of Theoretical Chemistry, IFM, Linköping University, SE-58183 Linköping, Sweden

‡Department of Chemistry, SRM University-AP, Mangalagiri-522240, Andhra Pradesh, India

§Jiangsu Key Laboratory for Chemistry of Low-Dimensional Materials, Jiangsu Engineering Laboratory for Environment Functional Materials, School of Chemistry and Chemical Engineering, Huaiyin Normal University, Huaian 223300, China

Supporting Information

Corresponding Authors

*Email: baswanthoruganti.s@srmap.edu.in (B.O.)

*Email: bodur@ifm.liu.se (B.D.)

Table of contents

Computational details	page S3
Figure S1	page S9
Figure S2	page S10
Figure S3	page S11
Figure S4	page S12
Figure S5	page S13
Figure S6	page S15
Figure S7	page S16
Figure S8	page S17
Table S1	page S18
Table S2	page S19
Table S3	page S20
Table S4	page S21
Cartesian coordinates and electronic energies of S_0 geometries of **1**	page S22
Cartesian coordinates and electronic energies of S_1 geometries of **1**	page S24
Cartesian coordinates and electronic energies of S_0 geometries of **2**	page S36
Cartesian coordinates and electronic energies of S_1 geometries of **2**	page S38
Cartesian coordinates and electronic energies of S_0 geometries of **3**	page S50
Cartesian coordinates and electronic energies of S_1 geometries of **3**	page S53
References	page S68

Computational details

Numerical integration in all density functional theory (DFT) and time-dependent DFT (TD-DFT)[1] calculations was carried out with default grid sizes (e.g., using the "Ultrafine" grid in the Gaussian 16 suite of programs[2] that comprises 99 radial shells and 590 angular points per shell). All calculations considered **1o**/**2o**/**3o** to adopt the so-called anti-parallel conformation,[3] which is the only conformation from which photocyclization can occur.[4]

Thermal electrocyclization and cycloreversion reactions. The S_0 geometries of **1o**/**1c**, **2o**/**2c** and **3o**/**3c** and the transition structures (**1ts**, **2ts** and **3ts**) that mediate both the thermal electrocyclization and the thermal cycloreversion reactions between the open and closed isomers were optimized with the B3LYP hybrid density functional in combination with the cc-pVTZ basis set and the SMD continuum solvation approach[5] to model an acetonitrile solvent, which has been used in previous experimental studies of **1o**.[3] Based on the resulting geometries, which are shown in Figure S1, frequency calculations were carried out at the same level of theory to obtain Gibbs free energies at room temperature and to ensure that the structures in question are either potential-energy minima with real vibrational frequencies only (**1o**/**1c**, **2o**/**2c** and **3o**/**3c**), or first-order saddle points with one imaginary vibrational frequency along the electrocyclization/cycloreversion reaction coordinate (**1ts**, **2ts** and **3ts**).

Vertical excitation energies. Based on the optimized S_0 geometries of **1o**–**3o**, vertical $S_0 \rightarrow S_1$ excitation energies were calculated by means of TD-DFT and the complete active space second-order perturbation theory (CASPT2) method,[6] employing in both cases the cc-pVTZ basis set. While the TD-DFT calculations were performed using an SMD-based description of acetonitrile, the CASPT2 calculations were carried out in the gas phase. The TD-DFT calculations were done within the Tamm-Dancoff approximation (TDA)[7] and using hybrid functionals of three different types: B3LYP (a global hybrid GGA), M06-2X[8] (a global hybrid meta-GGA) and ωB97X-D[9] (a range-separated hybrid GGA). The TDA was employed for all TD-DFT calculations in this work. The CASPT2 calculations, in turn, were done with the multi-state variant of this method (MS-CASPT2),[6b] and were based on complete active space self-consistent field (CASSCF)[10] wavefunctions computed with inclusion of the full π-systems of **1o**–**3o** in the active spaces, i.e., using CAS(14,14), CAS(12,12) and CAS(10,10) for **1o**, **2o** and **3o**, respectively (see Figure S2). The CASSCF wavefunctions for the S_1 state were computed by

means of state averaging (with equal weights) over the S_0, S_1 and S_2 states (for **1o** and **3o**), or over the S_0 and S_1 states (for **2o**).

Photocyclization reactions. Starting from the vertically excited S_1 Franck-Condon (FC) points of **1o**–**3o**, which occur at C1–C1' distances of 3.38 (**1o**), 3.61 (**2o**) and 3.58 Å (**3o**) (see Figure S1), the photocyclization reactions of **1o**/**2o**/**3o** into **1c**/**2c**/**3c** were modeled at the TD-B3LYP/cc-pVTZ/SMD level of theory by performing a series of constrained S_1 geometry optimizations in which the respective C1–C1' distance was varied in steps of 0.10 Å between 3.28 and 1.58 Å (**1o** → **1c** reaction), 3.51 and 1.61 Å (**2o** → **2c** reaction), and 3.48 and 1.58 Å (**3o** → **3c** reaction), respectively. In each optimization, carried out with analytic TD-DFT gradients,[11] all other geometric degrees of freedom than the C1–C1' distance were allowed to relax. From these calculations, the reactions of **1o** and **3o** were found to be barrierless, which implies that nearby minima are absent along the corresponding potential energy surfaces. The reaction of **2o**, on the other hand, was found to have a distinct barrier relative to a minimum (at C1–C1' = 3.55 Å, close to the FC point) that could be located through a fully unconstrained S_1 geometry optimization and characterized by means of a S_1 frequency calculation performed with the help of an analytic TD-DFT Hessian.[12] For comparative purposes, the S_1 energies of some of the resulting geometries were also determined through TD-DFT/cc-pVTZ/SMD (using M06-2X and ωB97X-D) and MS-CASPT2/cc-pVTZ singlepoint calculations (the corresponding CASSCF treatment was identical to that specified above).

As for the photocyclization reaction of **4o** – the diarylethene with a four-membered cyclobutene π-linker that was used as a reference compound to assess the potential impact of ring strain on the photocyclization of **2o** (which similarly features a four-membered cyclobutadiene π-linker) – this process was modeled using the same protocol that was adopted for the corresponding reactions of **1o**/**2o**/**3o**. Thus, starting from the S_1 FC point of **4o** at a C1–C1' distance of 3.49 Å, constrained S_1 geometry optimizations were performed at the TD-B3LYP/cc-pVTZ/SMD level of theory at C1–C1' distances of 3.39, 3.29, … 1.59 Å.

NAMD simulations. Starting from the respective S_1 FC point, the non-adiabatic molecular dynamics (NAMD) simulations[13] of the photocyclization reactions of **1o**–**3o** were performed with B3LYP (TD-B3LYP in the S_1 state) using Tully's fewest switches algorithm[14] as implemented in TURBOMOLE.[15] Because of the higher computational cost compared to the static calculations, for these simulations, the cc-pVTZ basis set was replaced with the smaller cc-pVDZ one. For each of the three reactions, the

simulations were run for maximally 300 fs with 25 different initial nuclear velocities generated randomly from a Maxwell-Boltzmann distribution at 300 K. For the classical propagation of the nuclei in Tully's method, the leapfrog Verlet algorithm[16] was used with a fixed integration time step of 1 fs. For the quantum mechanical description of the electrons in this method, the total wavefunction $\Psi(\mathbf{r}, t; \mathbf{R})$ is expressed as a linear combination of adiabatic electronic states $\{\phi_j(\mathbf{r}; \mathbf{R})\}$ with time-dependent complex amplitudes $\{a_j(t)\}$

$$\Psi(\mathbf{r}, t; \mathbf{R}) = \sum_j a_j(t)\phi_j(\mathbf{r}; \mathbf{R}), \tag{1}$$

where $\mathbf{r}$ denotes the electronic coordinates and $\mathbf{R} = \mathbf{R}(t)$ is the trajectory followed by the nuclei. In the present case, this expansion included the S_0 and S_1 states. The requirement that $\Psi(\mathbf{r}, t; \mathbf{R})$ satisfies the time-dependent Schrödinger equation then leads to a set of coupled differential equations that can be integrated numerically to obtain the amplitudes:

$$\mathrm{i}\hbar\dot{a_k} = a_k E_k(\mathbf{R}) - \mathrm{i}\hbar \sum_j a_j C_{kj}. \tag{2}$$

Here, $E_k(\mathbf{R})$ is the potential energy of the k-th adiabatic electronic state, and $\{C_{kj}\}$ are the non-adiabatic coupling matrix elements between states k and j, defined as

$$C_{kj} = \left\langle \phi_k(\mathbf{r}; \mathbf{R}) \middle| \frac{\partial}{\partial t} \middle| \phi_j(\mathbf{r}; \mathbf{R}) \right\rangle, \tag{3}$$

where the integration is over the electronic coordinates $\mathbf{r}$.

In the TURBOMOLE implementation of Tully's method,[15] the probability for a hop/transition between states k and j is computed based on the population $|a_n(t)|^2$ of the states and the non-adiabatic coupling C_{kj} between them. The computed transition probability is compared with a random number between 0 and 1, and a hop is invoked if the probability is larger than that random number. However, in order to avoid problems with imaginary excitation energies in TD-DFT,[17] a hop is always enforced if the corresponding energy gap is smaller than 0.2 eV, regardless of the value of the transition probability.

In order to monitor the time evolution of the photocyclizations, at any given time during the NAMD simulations the classical populations of the S_1 and S_0 states were evaluated as (following the usual convention) the fractions of trajectories evolving in the respective state at that particular time:

$$P_j(t) = \frac{N_j(t)}{N_{\text{trajs}}} . \tag{4}$$

In order to classify any trajectory for **1o**–**3o** as successful in completing the corresponding photocyclization within 300 fs, it was required that the trajectory decays to (and then remains in) the S_0 state and that the average C1–C1' distance during the last 25 fs of the simulation does not exceed 1.70 Å (a value that simultaneously reflects proximity to the ring-closed isomer and allows for dynamical fluctuations). The photocyclization time for each successful trajectory was taken to be the time at which a C1–C1' distance of 1.70 Å was first reached in the corresponding simulation, and the average photocyclization times for **1o** and **3o** were obtained by averaging these values over all successful trajectories in the respective case (for **2o**, no successful trajectories were observed).

Aromaticity indices. The nucleus-independent chemical shift (NICS) indices[18] for the benzene/cyclobutadiene/cyclohexene π-linkers along the photocyclization paths of **1o**/**2o**/**3o** were calculated from state-specific CASSCF/cc-pVDZ S_0 and S_1 wavefunctions of the different diarylethene species at their B3LYP/cc-pVTZ/SMD S_0 and TD-B3LYP/cc-pVTZ/SMD S_1 geometries, respectively. These indices are of magnetic origin and probe ring currents induced by circulating π-electrons. Specifically, employing gauge-including atomic orbitals and (12,12)/(12,12)/(10,10) active spaces for species along the paths of **1o**/**2o**/**3o**, a NICS-scan procedure[19] was used to calculate so-called $NICS_{zz}$ values (corresponding to the zz-component of the magnetic shielding tensor[18b]) at distances above the geometric centers of the π-linkers ranging from 1.50 to 2.00 Å in steps of 0.10 Å. Such a procedure alleviates the arbitrariness introduced by, alternatively, having to choose one specific distance at which to calculate a single $NICS_{zz}$ value.[19] Furthermore, although it is not uncommon for shorter distances to be considered,[19c] the current distances were chosen so as to simultaneously minimize both contributions from σ-electrons to the induced magnetic field[19b] and the offset between the center of the field and the normal axis passing through the geometric center of the π-linker.[19d,19e]

Besides calculating NICS indices, it was deemed worthwhile to also obtain data based on an electronic aromaticity index. To this end, Shannon aromaticity (SA) indices[20] for the π-linkers were calculated from the B3LYP/cc-pVTZ/SMD S_0 and TD-B3LYP/cc-pVTZ/SMD S_1 electron densities of

the different diarylethene species at their correspondingly optimized geometries. This index is based on Bader's theory of atoms in molecules[21] and measures the variation in electron density at bond critical points (BCPs) of the ring system at hand. Specifically, it is formulated in terms of the Shannon entropy[22]

$$S = -\sum_i^N p_i(\mathbf{r}_\mathrm{c}) \ln p_i\,(\mathbf{r}_\mathrm{c}), \tag{5}$$

where $\mathbf{r}_\mathrm{c}$ is a BCP, N is the number of BCPs, and $p_i(\mathbf{r}_\mathrm{c})$ is the normalized probability electron density at a given BCP. With this definition, the SA index is calculated as the difference between the Shannon entropy that the system of interest would have if it was perfectly aromatic, and the Shannon entropy that it actually has. Accordingly, a small SA value reflects a small variation in electron density at BCPs, which is a key characteristic of an aromatic system.[20]

In order to verify that the calculated SA values for any given π-linker are sensible, geometric tests were performed where the SA values pertaining to the S_0 state were compared with the values for the maximum absolute deviation (MAD) between the S_0 (B3LYP/cc-pVTZ/SMD) carbon-carbon bond lengths of that particular π-linker, and where, similarly, the SA values for the S_1 state were compared with the MAD values for the S_1 (TD-B3LYP/cc-pVTZ/SMD) carbon-carbon bond lengths. Then, if the SA values are indeed sensible, one expects small/large SA values to be correlated with small/large MAD values, which is also what was observed (see Figure S5a). In this light, it should be noted that an established geometric aromaticity index is available in the form of the harmonic oscillator model of aromaticity (HOMA) index,[23] which probes the deviation of the heavy-atom bond lengths of the ring in question (R_i) from an optimum aromatic reference value (R_opt). Specifically, this index is defined as

$$\mathrm{HOMA} = 1 - \frac{\alpha}{n}\sum_i^n (R_i - R_\mathrm{opt})^2, \tag{6}$$

where n is the number of carbon-carbon bonds in the ring and α is an empirical normalization factor chosen such that the HOMA value approaches 1 (0) for an aromatic (non-aromatic) compound. However, the reason for using MAD instead of HOMA values to verify the soundness of the SA values is that no choice of reference parameters is needed to calculate MAD values that provide a balanced assessment of aromaticity in the benzene, cyclobutadiene and cyclohexene π-linkers. For the calculation of HOMA values, on the other hand, it would have been difficult to select reference parameters (R_opt and α) that are suitable in this regard. At any rate, using the standard parameters[23]

$\alpha = 257.7$ Å^{-2} and $R_{\text{opt}} = 1.388$ Å to calculate HOMA values for the benzene π-linker based on S_0 (B3LYP/cc-pVTZ/SMD) and S_1 (TD-B3LYP/cc-pVTZ/SMD) geometries, it can be deduced from a comparison of the resulting data with the corresponding MAD values in Figure S5b that, as far as this system goes, the basic MAD measure is consistent with the more elaborate HOMA one. More precisely, smaller MAD values are associated with HOMA values closer to 1.

Software used. The calculations were done with the Gaussian 16,[2] OpenMolcas 18.09,[24] TURBOMOLE 7.4,[15b,25] Dalton 2016.2,[26] and Multiwfn 3.7[27] suites of programs. Gaussian 16 was used for DFT and TD-DFT calculations. OpenMolcas 18.09 was used for CASSCF and CASPT2 calculations. TURBOMOLE 7.4 was used for NAMD simulations. Dalton 2016.2 was used for NICS-index calculations. Multiwfn 3.7 was used for HOMA- and SA-index calculations.

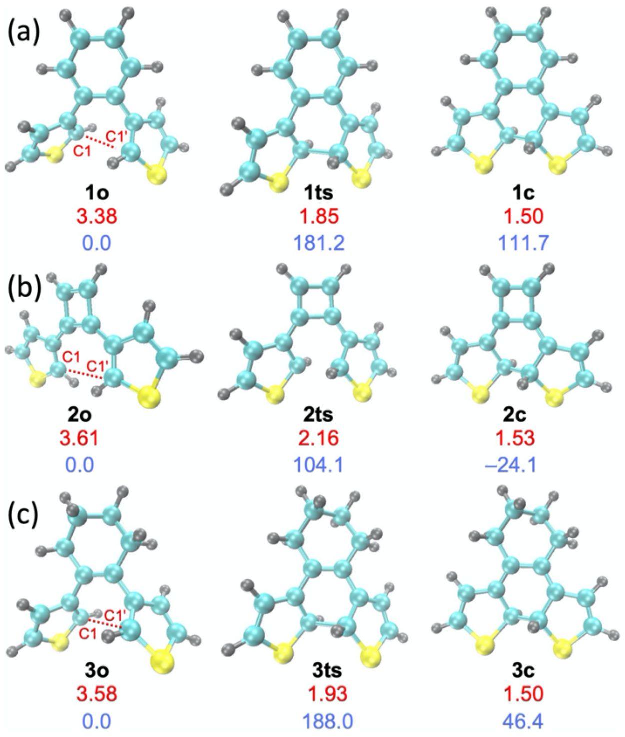

Figure S1. Calculated stationary points for the thermal electrocyclization and cycloreversion reactions of **1o** (a), **2o** (b) and **3o** (c). Values in red font are the corresponding C1–C1' distances (in Å) and values in blue font are the corresponding relative free energies (in kJ mol^{-1}).

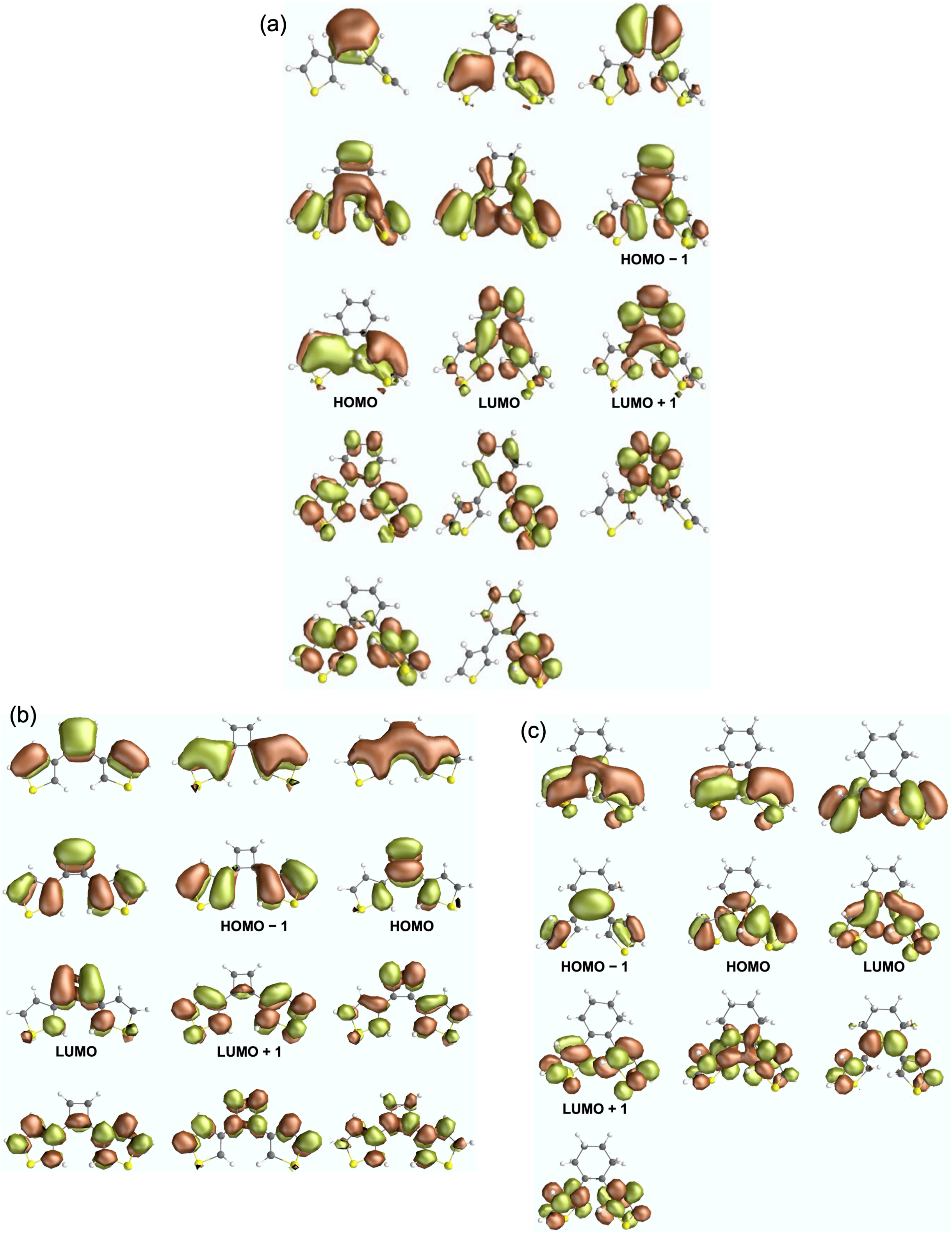

Figure S2. CASSCF/cc-pVTZ natural orbitals in the S_0 state of **1o** (a), **2o** (b) and **3o** (c) calculated based on B3LYP/cc-pVTZ/SMD S_0 geometries.

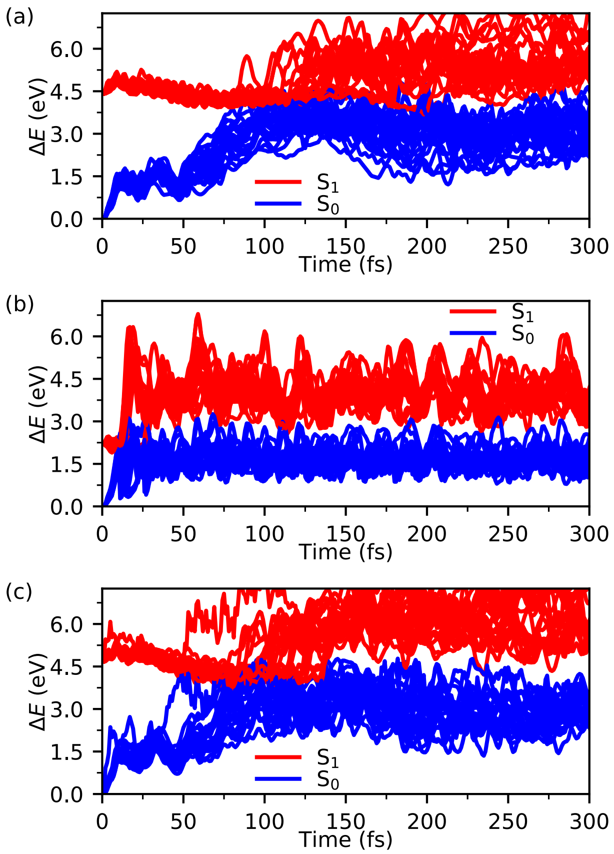

Figure S3. Changes in S_1 and S_0 energies (ΔE) during the 25 NAMD trajectories run for each of **1o** (a), **2o** (b) and **3o** (c).

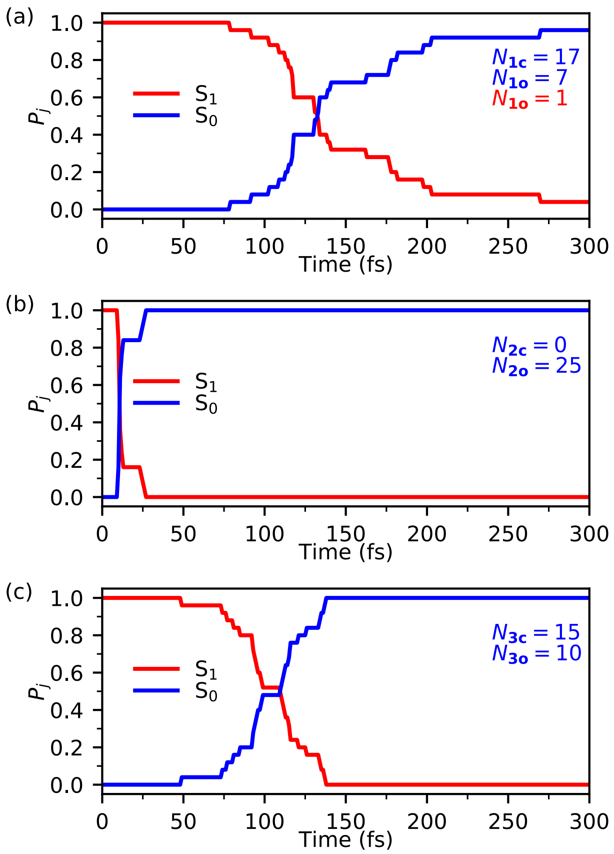

Figure S4. Time evolution of the classical populations (P_j) of the S_1 and S_0 states during the 25 NAMD trajectories run for each of **1o** (a), **2o** (b) and **3o** (c). The numbers of trajectories that produce the ring-closed and ring-open isomers after hopping to the S_0 state are indicated in blue font as $N_{\mathbf{1c}}/N_{\mathbf{2c}}/N_{\mathbf{3c}}$ and $N_{\mathbf{1o}}/N_{\mathbf{2o}}/N_{\mathbf{3o}}$, respectively. For **1o**, one of the trajectories remained in the S_1 state for the entirety of the simulation without undergoing any ring-closing. This trajectory is indicated in red font as $N_{\mathbf{1o}}$.

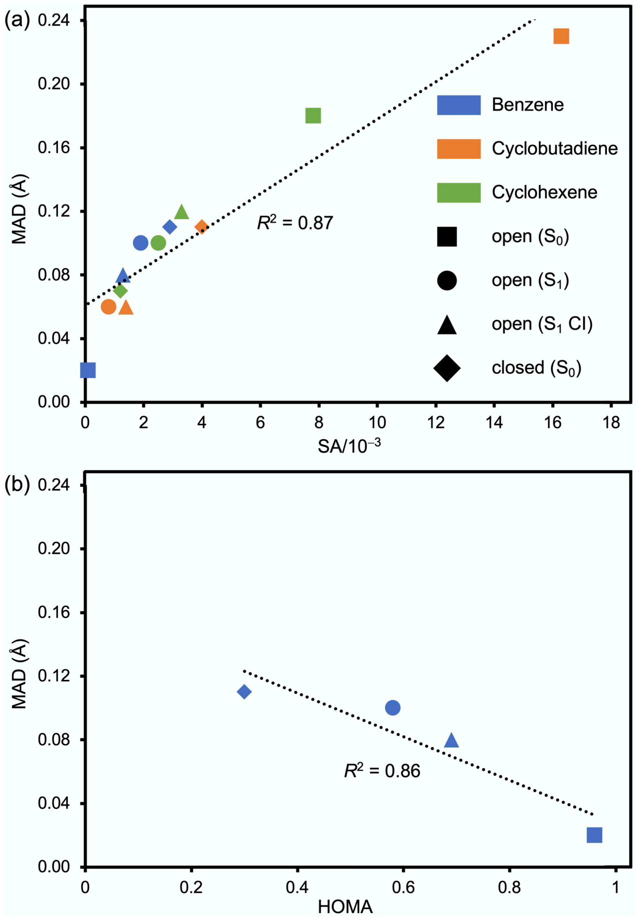

Figure S5. (a) Correlation between MAD and SA values for the benzene/cyclobutadiene/cyclohexene π-linkers of **1o**/**2o**/**3o** calculated at key points along the corresponding photocyclization paths. (b) Correlation between MAD and HOMA values for the benzene π-linker of **1o** calculated at key points along the corresponding photocyclization path.

Discussion of the results in Figure S5. The magnetic $NICS_{zz}$ indices in Figure 3 of the main text offer a clear picture of how the aromatic character of the π-linkers of **1o**/**2o**/**3o** evolves along the photocyclization paths. Pleasingly, this picture is corroborated by the calculation of electronic SA indices (see Figure S6). The choice to complement the analysis with this particular type of aromaticity index is informed by the results in Figure S5a, which show that the SA values for the π-linkers are sensibly correlated with the corresponding geometric MAD values, both qualitatively (small SA values are associated with small MAD values) and quantitatively (R^2 value of 0.87). Here, the reason for excluding the S_1 FC point in the analysis is that a geometric index cannot probe changes in aromaticity

from a vertical photoexcitation. In support of the use of the basic MAD measure to corroborate the SA values, calculations summarized in Figure S5b show that the correlation between MAD and HOMA values for the benzene π-linker of **1o**, to which the HOMA index is ideally applicable, follows the expected trend, with smaller MAD values being associated with HOMA values closer to 1.

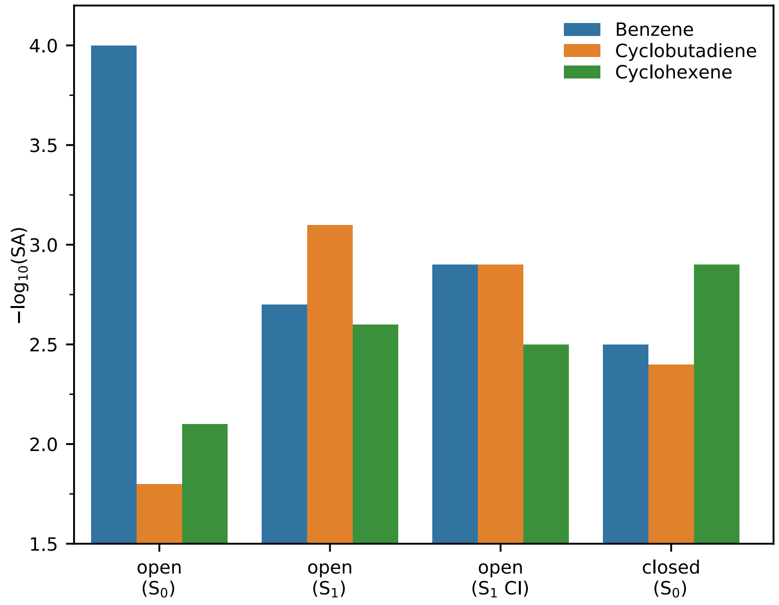

Figure S6. Negative logarithms of the SA values for the benzene/cyclobutadiene/cyclohexene π-linkers of **1o**/**2o**/**3o** calculated at key points along the corresponding photocyclization paths. The points are presented from left to right in the order in which they appear along the paths (see Table S4 for the corresponding C1–C1' distances).

Discussion of the results in Figure S6. The key findings in Figure 3 of the main text concern the distinct difference in how the aromatic character of the benzene and cyclobutadiene π-linkers of **1o** and **2o**, respectively, changes upon photoexcitation, and the relative lack of such changes for the non-aromatic cyclohexene π-linker of **3o**. Qualitatively, these $NICS_{zz}$-based findings are supported by the SA values in Figure S6, presented using a scale in which a larger $-\log_{10}(SA)$ value (i.e., a smaller SA value) signifies a more aromatic system. Consistently with the results in Figure 3, Figure S6 shows that the changes in the aromatic character of the benzene and cyclobutadiene π-linkers in the S_1 state of **1o** and **2o** occur in polar opposite directions. Specifically, the former experiences a marked loss of aromaticity relative to the S_0 state (the $-\log_{10}(SA)$ value decreases from 4.0 to below 3), whereas the latter experiences a distinct gain (the $-\log_{10}(SA)$ value increases from 1.8 to ~3). For the cyclohexene π-linker of **3o**, on the other hand, the small $-\log_{10}(SA)$ value of 2.1 shown by the S_0 state is quite well retained in S_1, changing (increasing) by no more than ~0.5. Qualitatively, this is consistent with the corresponding changes in the $NICS_{zz}$ values in Figure 3 being much smaller than those for the π-linkers of **1o** and **2o**.

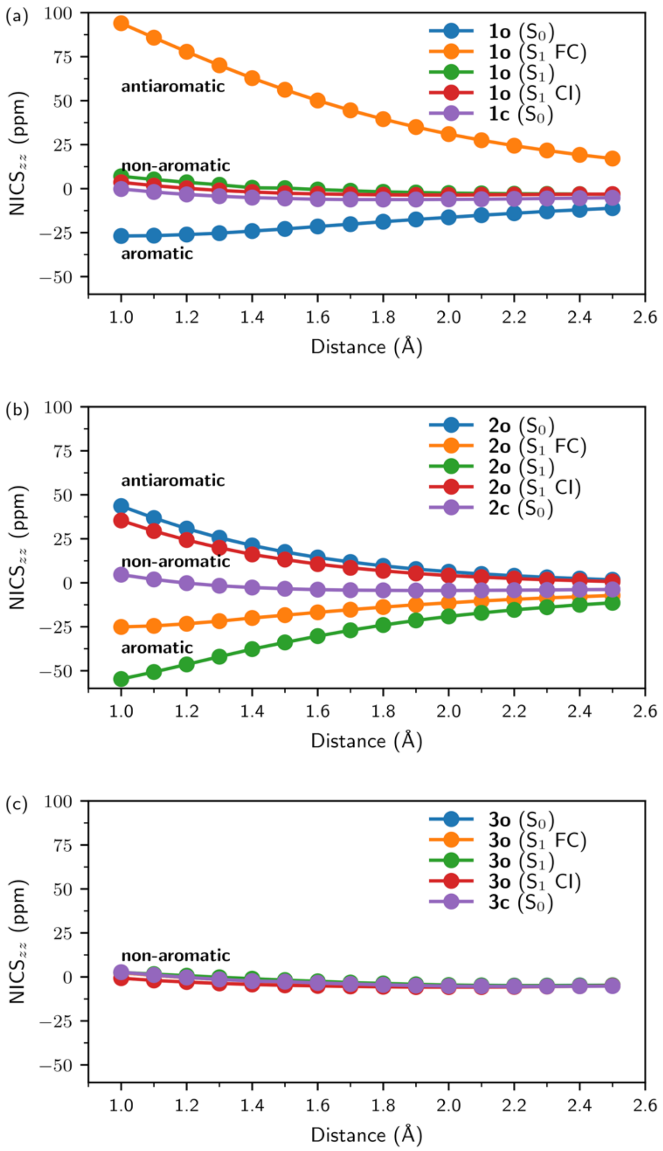

Figure S7. NICS$_{zz}$ values for the benzene/cyclobutadiene/cyclohexene π-linkers of **1o**/**2o**/**3o** calculated at key points along the corresponding photocyclization paths and at a wider range of different distances above the geometric ring centers than considered in the main text (1.00–2.50 Å instead of 1.50–2.00 Å).

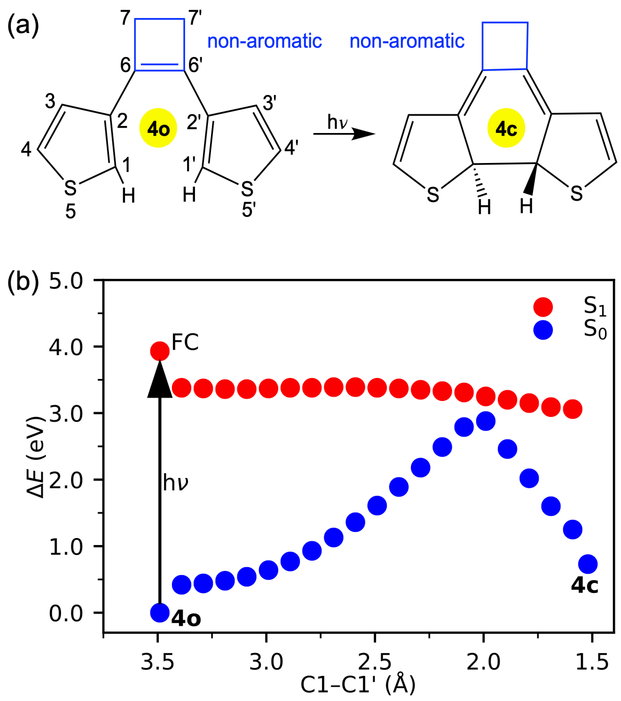

Figure S8. (a) Photocyclization reaction of **4o** into **4c**. (b) Calculated photocyclization path in the S_1 state of **4o** with energies (ΔE) given relative to the S_0 energy of **4o**. Shown are also the S_0 energies at the S_1 geometries along the photocyclization path.

Table S1. Calculated Free Energies (kJ mol^{-1}) and Atomic Spin Densities at the Transition Structures for the Thermal Electrocyclization and Cycloreversion Reactions of 1o–3o[a]

				spin density[b]						
reaction	$\Delta G_{o\to c}$[c]	$\Delta G^{\ddagger}_{o\to c}$[d]	$\Delta G^{\ddagger}_{c\to o}$[e]	C1	C1'	C2	C2'	C6	C6'	$\langle S^2\rangle$[f]
**1o** ↔ **1c**	111.7	181.2	69.5	−0.17	0.17	0.35	−0.35	−0.11	0.11	0.68
**2o** ↔ **2c**	−24.1	104.1	128.2	0.00	0.00	0.00	0.00	0.00	0.00	0.00
**3o** ↔ **3c**	46.4	188.0	141.6	−0.24	0.24	0.38	−0.38	−0.27	0.27	0.87

[a]All calculations carried out at the B3LYP/cc-pVTZ/SMD level of theory. For the closed-shell species **1o**/**2o**/**3o** and **1c**/**2c**/**3c**, all spin densities are exactly zero. [b]Spin densities are only given for the reactive carbon atoms (see Scheme 1). [c]Reaction free energy for electrocyclization. [d]Free-energy barrier for electrocyclization. [e]Free-energy barrier for cycloreversion. [f]Expectation value of the spin-squared operator.

Table S2. Vertical Excitation Energies (VEEs) and FC Relaxation Energies (FCREs) for 1o–3o Calculated with Different Density Functionals (eV)[a]

species	functional	VEE($S_0 \rightarrow S_1$)[b]	FCRE(S_1)
**1o**	B3LYP	4.43 (0.09)	1.55[c]
	M06-2X	4.98 (0.15)	1.73[c]
	ωB97X-D	5.02 (0.14)	1.71[c]
**2o**	B3LYP	2.12 (0.16)	0.77[d]
	M06-2X	2.23 (0.14)	0.68[d]
	ωB97X-D	2.24 (0.14)	0.68[d]
**3o**	B3LYP	4.55 (0.12)	1.68[e]
	M06-2X	5.11 (0.23)	1.99[e]
	ωB97X-D	5.13 (0.23)	1.94[e]

[a]TD-DFT/cc-pVTZ/SMD calculations. [b]Calculated using S_0 geometries optimized at the B3LYP/cc-pVTZ/SMD level of theory. Oscillator strengths given in parentheses. [c]Calculated as the decrease in S_1 energy at C1–C1' = 1.78 Å along the photocyclization path in Figure 1 of the main text relative to the FC point at 3.38 Å. [d]Calculated as the decrease in S_1 energy at the TD-B3LYP/cc-pVTZ/SMD S_1 equilibrium geometry (with C1–C1' = 3.55 Å) relative to the FC point at 3.61 Å. [e]Calculated as the decrease in S_1 energy at C1–C1' = 1.98 Å along the photocyclization path in Figure 1 of the main text relative to the FC point at 3.58 Å.

Table S3. Vertical Excitation Energies (VEEs) and FC Relaxation Energies (FCREs) for 1o–3o Calculated with the MS-CASPT2 Method (eV)[a]

species	active space	VEE($S_0 \rightarrow S_1$)[b]	FCRE(S_1)	CASSCF occupation numbers for active molecular orbitals in the S_0 and S_1 states							
**1o**	(14,14)	4.73	1.33[c]	S_0	1.96	1.94	1.90	1.91	1.91	1.89	1.94
					0.11	0.11	0.09	0.09	0.04	0.06	0.06
				S_1	1.86	1.90	1.44	1.94	1.91	1.45	1.94
					0.58	0.56	0.10	0.09	0.05	0.06	0.11
**2o**	(12,12)	2.51	1.04[d]	S_0	1.92	1.94	1.95	1.90	1.90	1.76	
					0.24	0.10	0.10	0.06	0.08	0.04	
				S_1	1.90	1.93	1.94	1.80	1.81	0.55	
					1.45	0.20	0.20	0.06	0.10	0.05	
**3o**	(10,10)	5.19	2.30[e]	S_0	1.94	1.94	1.91	1.92	1.90		
					0.10	0.10	0.06	0.08	0.06		
				S_1	1.92	1.80	1.54	1.77	1.43		
					0.27	0.65	0.07	0.37	0.16		

[a]MS-CASPT2/cc-pVTZ calculations in the gas phase. [b]Calculated using S_0 geometries optimized at the B3LYP/cc-pVTZ/SMD level of theory. [c]Calculated as the decrease in S_1 energy at C1–C1' = 1.78 Å along the photocyclization path in Figure 1 of the main text relative to the FC point at 3.38 Å. [d]Calculated as the decrease in S_1 energy at the TD-B3LYP/cc-pVTZ/SMD S_1 equilibrium geometry (with C1–C1' = 3.55 Å) relative to the FC point at 3.61 Å. [e]Calculated as the decrease in S_1 energy at C1–C1' = 1.98 Å along the photocyclization path in Figure 1 of the main text relative to the FC point at 3.58 Å.

Discussion of the results in Tables S2 and S3. Two key features of the B3LYP-calculated photocyclization paths in Figure 1 of the main text are the vertical $S_0 \rightarrow S_1$ excitation energies of the **1o**/**2o**/**3o** species and the energy differences between their S_1 FC points and subsequent points along their photocyclization paths, which we here term FC relaxation energies (FCREs). In Tables S2 and S3, the B3LYP values of these quantities are compared with values obtained from calculations using both the M06-2X and ωB97X-D density functionals and the MS-CASPT2. method. Encouragingly, this comparison reveals a clear qualitative agreement between the B3LYP data on the one hand and the M06-2X, ωB97X-D and MS-CASPT2 data on the other. For example, while B3LYP yields a FCRE of 1.55 eV for **1o**, the corresponding M06-2X, ωB97X-D and MS-CASPT2 estimates are 1.73, 1.71 and 1.33 eV, respectively. A further illustration of this point is the relatively small spread among the vertical $S_0 \rightarrow S_1$ excitation energies calculated for **1o**, which range from 4.43 (B3LYP) to 4.73 (MS-CASPT2), 4.98 (M06-2X) and 5.02 eV (ωB97X-D).

Table S4. NICS$_{zz}$ Values (ppm) for the Benzene/Cyclobutadiene/Cyclohexene π-Linkers of 1o/2o/3o Calculated at Key Points along the Corresponding Photocyclization Paths and at Different Distances (Å) Above the Geometric Ring Centers[a]

species/point	C1–C1' distance (Å)	π-linker	distance above the geometric ring center 1.50	1.60	1.70	1.80	1.90	2.00	<NICS$_{zz}$>[b]
**1o** (S_0)	3.38	benzene	−22.9	−21.5	−20.2	−18.8	−17.5	−16.3	−19.5
**1o** (S_1 FC)	3.38	benzene	56.2	50.1	44.5	39.5	35.0	31.0	42.7
**1o** (S_1)	2.68	benzene	0.3	−0.5	−1.2	−1.8	−2.2	−2.5	−1.3
**1o** (S_1 CI)	1.78	benzene	−2.6	−3.0	−3.3	−3.5	−3.6	−3.6	−3.3
**1c** (S_0)	1.50	benzene	−5.6	−6.0	−6.2	−6.2	−6.2	−6.1	−6.1
**2o** (S_0)	3.61	cyclobutadiene	17.5	14.4	11.8	9.6	7.8	6.3	11.2
**2o** (S_1 FC)	3.61	cyclobutadiene	−18.4	−16.8	−15.3	−13.8	−12.5	−11.4	−14.7
**2o** (S_1)[c]	3.55	cyclobutadiene	−33.9	−30.3	−27.0	−24.0	−21.4	−19.1	−26.0
**2o** (S_1 CI)	2.51	cyclobutadiene	13.2	10.6	8.5	6.8	5.3	4.1	8.1
**2c** (S_0)	1.53	cyclobutadiene	−3.4	−3.9	−4.2	−4.3	−4.4	−4.4	−4.1
**3o** (S_0)	3.58	cyclohexene	−2.7	−3.3	−3.8	−4.2	−4.4	−4.7	−3.8
**3o** (S_1 FC)	3.58	cyclohexene	−3.0	−3.6	−4.0	−4.4	−4.7	−4.9	−4.1
**3o** (S_1)	2.88	cyclohexene	−1.8	−2.5	−3.2	−3.7	−4.2	−4.6	−3.3
**3o** (S_1 CI)	1.98	cyclohexene	−4.8	−5.1	−5.4	−5.6	−5.8	−5.8	−5.4
**3c** (S_0)	1.50	cyclohexene	−2.7	−3.4	−4.0	−4.5	−4.9	−5.2	−4.1

[a]State-specific CASSCF/cc-pVDZ calculations in the gas phase based on B3LYP/cc-pVTZ/SMD S_0 and TD−B3LYP/cc-pVTZ/SMD S_1 geometries, and performed using (12,12), (12,12) and (10,10) active spaces for **1**, **2** and **3**, respectively. [b]The mean NICS$_{zz}$ value over the different distances above the geometric ring center. [c]The S_1 equilibrium geometry.

Cartesian coordinates (in Å) and electronic energies (E, in Ha) of S_0 geometries of 1

Geometries optimized at the B3LYP/cc-pVTZ/SMD level of theory.

1o

$E(S_0) = -1336.1398543$ Ha (all vibrational frequencies real)

C	0.705129	1.283255	−0.022194
C	−0.706339	1.282572	0.022052
C	1.375079	2.513089	−0.065968
C	−1.377386	2.511849	0.065837
C	0.692102	3.720225	−0.038583
C	−0.695474	3.719575	0.038467
C	−1.532740	0.050559	−0.010185
C	−1.408865	−0.957211	−0.928425
C	−2.614954	−0.195494	0.897171
C	1.532691	0.051991	0.010093
C	2.613701	−0.194217	−0.898626
C	3.267714	−1.363393	−0.658900
H	2.873909	0.474835	−1.706198
C	1.410972	−0.954803	0.929664
S	2.587683	−2.193592	0.694922
C	−3.267689	−1.365445	0.657793
S	−2.584996	−2.196618	−0.694096
H	−2.876876	0.474204	1.703647
H	−0.701014	−1.038248	−1.736625
H	−4.100164	−1.790867	1.194295
H	0.704283	−1.035481	1.738924
H	4.099755	−1.788727	−1.196147
H	2.456660	2.515667	−0.096779
H	1.241162	4.652156	−0.066603
H	−1.245391	4.651004	0.066507
H	−2.458964	2.513517	0.096576

1c

$E(S_0) = -1336.1015543$ Ha (all vibrational frequencies real)

C	−1.400468	0.734228	−0.021987
C	−1.400468	−0.734228	0.021987
C	−2.672456	1.403440	−0.115827
C	−2.672456	−1.403440	0.115827
C	−3.838872	0.714091	−0.069723
C	−3.838872	−0.714091	0.069723
H	−4.783057	1.239790	−0.125968
H	−4.783057	−1.239790	0.125968
H	−2.688480	−2.481234	0.200742
H	−2.688480	2.481234	−0.200742

C	−0.207430	−1.420837	−0.080377
C	1.068337	−0.650686	−0.373307
C	0.038871	−2.827575	−0.017026
C	−0.207430	1.420837	0.080377
C	0.038871	2.827575	0.017026
C	1.346251	3.167049	−0.018781
H	−0.744618	3.569969	−0.030507
C	1.068337	0.650686	0.373307
S	2.458917	1.811139	−0.010788
C	1.346251	−3.167049	0.018781
S	2.458917	−1.811139	0.010788
H	−0.744618	−3.569969	0.030507
H	1.120986	0.454041	1.454670
H	1.120986	−0.454041	−1.454670
H	1.747093	4.168080	−0.069189
H	1.747093	−4.168080	0.069189

1ts

$E(S_0)$ = −1336.0715619 Ha (one imaginary vibrational frequency)

C	1.445627	0.704063	−0.106881
C	1.445627	−0.704063	0.106881
C	2.665488	1.369136	−0.217858
C	2.665488	−1.369136	0.217858
C	3.876271	0.684871	−0.108511
C	3.876271	−0.684871	0.108511
H	4.809097	1.225987	−0.196659
H	4.809097	−1.225987	0.196659
H	2.670193	−2.434294	0.409128
H	2.670193	2.434294	−0.409128
C	0.164514	−1.417612	0.178541
C	−1.044952	−0.719005	0.580414
C	−0.098138	−2.661398	−0.410853
C	0.164514	1.417612	−0.178541
C	−0.098138	2.661398	0.410853
C	−1.422047	3.032616	0.402151
H	0.666081	3.262326	0.884145
C	−1.044952	0.719005	−0.580414
S	−2.419850	1.854916	−0.399258
C	−1.422047	−3.032616	−0.402151
S	−2.419850	−1.854916	0.399258
H	0.666081	−3.262326	−0.884145
H	−1.083809	0.185734	−1.522410
H	−1.083809	−0.185734	1.522410
H	−1.864538	3.908182	0.848582
H	−1.864538	−3.908182	−0.848582

Cartesian coordinates (in Å) and electronic energies (E, in Ha) of S_1 geometries of 1

Geometries optimized at the TD-B3LYP/cc-pVTZ/SMD level of theory with all geometric degrees of freedom relaxed except the C1–C1' distance.

C1–C1' = 3.28 Å

$E(S_1)$ = −1336.00576560 Ha

C	−0.696173	1.151965	0.254375
C	0.696110	1.151958	−0.254319
C	−1.222080	2.399772	0.681317
C	1.222006	2.399739	−0.681350
C	−0.601193	3.586929	0.381824
C	0.601165	3.586913	−0.381831
C	1.584258	0.042444	−0.097108
C	1.418661	−1.025607	0.819889
C	2.843780	−0.101921	−0.791775
C	−1.584304	0.042447	0.097102
C	−2.843881	−0.101926	0.791673
C	−3.550397	−1.196714	0.435676
H	−3.184921	0.579140	1.556435
C	−1.418590	−1.025666	−0.819808
S	−2.734518	−2.124186	−0.802797
C	3.550372	−1.196653	−0.435757
S	2.734632	−2.124074	0.802846
H	3.184714	0.579110	−1.556618
H	0.624598	−1.144452	1.537708
H	4.495864	−1.537564	−0.825595
H	−0.624494	−1.144507	−1.537590
H	−4.495907	−1.537636	0.825461
H	−2.194363	2.424280	1.154470
H	−1.055130	4.523759	0.676007
H	1.055137	4.523733	−0.675995
H	2.194279	2.424215	−1.154527

C1–C1' = 3.18 Å

$E(S_1)$ = −1336.00768845 Ha

C	−0.701069	1.176620	0.237738
C	0.700970	1.176644	−0.237691
C	−1.244543	2.425102	0.638676
C	1.244371	2.425139	−0.638684
C	−0.615435	3.612477	0.358145
C	0.615223	3.612495	−0.358166
C	1.572555	0.054329	−0.084537
C	1.369457	−1.031653	0.805104
C	2.840653	−0.096998	−0.759254

C	−1.572602	0.054267	0.084543
C	−2.840754	−0.097110	0.759146
C	−3.521029	−1.212821	0.416085
H	−3.206958	0.594468	1.502610
C	−1.369312	−1.031782	−0.804972
S	−2.667354	−2.153188	−0.787673
C	3.521038	−1.212644	−0.416200
S	2.667556	−2.152992	0.787707
H	3.206748	0.594575	−1.502776
H	0.570420	−1.142619	1.518135
H	4.466501	−1.562338	−0.798247
H	−0.570144	−1.142781	−1.517853
H	−4.466504	−1.562563	0.798059
H	−2.231901	2.448481	1.079561
H	−1.080976	4.549339	0.633491
H	1.080719	4.549370	−0.633539
H	2.231724	2.448549	−1.079575

C1–C1' = 3.08 Å

$E(S_1)$ = −1336.00950877 Ha

C	−0.705622	1.201418	0.220172
C	0.705566	1.201456	−0.220129
C	−1.266110	2.450310	0.594332
C	1.265981	2.450373	−0.594320
C	−0.628838	3.637807	0.333553
C	0.628626	3.637836	−0.333590
C	1.560309	0.066448	−0.070832
C	1.319442	−1.035489	0.791335
C	2.835176	−0.094149	−0.727134
C	−1.560305	0.066367	0.070868
C	−2.835202	−0.094266	0.727100
C	−3.488043	−1.231071	0.398684
H	−3.225791	0.605990	1.449829
C	−1.319344	−1.035575	−0.791263
S	−2.598068	−2.181211	−0.771569
C	3.488099	−1.230902	−0.398706
S	2.598185	−2.181102	0.771545
H	3.225702	0.606113	−1.449893
H	0.518408	−1.135576	1.503216
H	4.431930	−1.590998	−0.775045
H	−0.518248	−1.135656	−1.503075
H	−4.431874	−1.591205	0.774988
H	−2.267668	2.472641	1.002056
H	−1.105066	4.574762	0.589650
H	1.104794	4.574814	−0.589715
H	2.267539	2.472755	−1.002040

C1–C1' = 2.98 Å

$E(S_1)$ = −1336.01120831 Ha

C	−0.709683	1.225473	0.202289
C	0.709772	1.225414	−0.202348
C	−1.285819	2.474491	0.549600
C	1.286038	2.474389	−0.549592
C	−0.640873	3.662106	0.308689
C	0.641188	3.662056	−0.308687
C	1.547845	0.078355	−0.056710
C	1.269171	−1.037979	0.777555
C	2.827943	−0.093286	−0.696204
C	−1.547843	0.078478	0.056689
C	−2.827909	−0.093094	0.696269
C	−3.453398	−1.249989	0.383020
H	−3.241657	0.614021	1.399273
C	−1.269299	−1.037856	−0.777624
S	−2.528581	−2.207291	−0.754744
C	3.453345	−1.250221	−0.382926
S	2.528368	−2.207506	0.754721
H	3.241793	0.613829	−1.399149
H	0.470489	−1.124229	1.493422
H	4.394640	−1.621271	−0.754906
H	−0.470721	−1.124091	−1.493611
H	−4.394686	−1.620991	0.755065
H	−2.300507	2.495682	0.923712
H	−1.126988	4.599186	0.545034
H	1.127416	4.599099	−0.544946
H	2.300752	2.495495	−0.923641

C1–C1' = 2.88 Å

$E(S_1)$ = −1336.01276490 Ha

C	−0.713601	1.249318	0.183060
C	0.713644	1.249306	−0.183073
C	−1.305110	2.498361	0.501925
C	1.305184	2.498335	−0.501924
C	−0.652730	3.686094	0.282242
C	0.652825	3.686083	−0.282250
C	1.535309	0.090577	−0.041173
C	1.218477	−1.038933	0.764517
C	2.819172	−0.093300	−0.665299
C	−1.535290	0.090607	0.041185
C	−2.819144	−0.093240	0.665341
C	−3.416081	−1.270389	0.369408
H	−3.255973	0.619484	1.348504
C	−1.218523	−1.038892	−0.764549

S	−2.457392	−2.233069	−0.735964
C	3.416047	−1.270490	−0.369402
S	2.457316	−2.233142	0.735958
H	3.256042	0.619421	−1.348439
H	0.428306	−1.108429	1.491200
H	4.353544	−1.653258	−0.739109
H	−0.428382	−1.108395	−1.491264
H	−4.353590	−1.653125	0.739118
H	−2.332315	2.518273	0.840417
H	−1.148269	4.623291	0.497688
H	1.148384	4.623271	−0.497687
H	2.332401	2.518225	−0.840382

C1–C1' = 2.78 Å

$E(S_1)$ = −1336.01420437 Ha

C	−0.716977	1.272438	0.162973
C	0.717339	1.272260	−0.163055
C	−1.322491	2.521468	0.452787
C	1.323180	2.521152	−0.452774
C	−0.662999	3.709246	0.254954
C	0.663968	3.709087	−0.254952
C	1.523075	0.102433	−0.024773
C	1.167623	−1.039333	0.750995
C	2.809678	−0.094450	−0.634417
C	−1.523013	0.102811	0.024756
C	−2.809573	−0.093777	0.634588
C	−3.378172	−1.290333	0.357379
H	−3.268996	0.623565	1.297953
C	−1.167995	−1.038982	−0.751173
S	−2.386905	−2.257321	−0.715089
C	3.377913	−1.291179	−0.357212
S	2.386231	−2.257976	0.715043
H	3.269389	0.622811	−1.297671
H	0.390821	−1.091250	1.493306
H	4.311123	−1.685503	−0.725637
H	−0.391373	−1.091015	−1.493666
H	−4.311437	−1.684429	0.725908
H	−2.361285	2.540547	0.754210
H	−1.166954	4.646628	0.449116
H	1.168162	4.646348	−0.449076
H	2.361991	2.539986	−0.754155

C1–C1' = 2.68 Å

$E(S_1)$ = −1336.01558511 Ha

C	0.719782	1.294236	−0.142973

C	−0.720544	1.293796	0.143127
C	1.337199	2.543218	−0.405352
C	−1.338745	2.542403	0.405449
C	0.671296	3.730975	−0.228949
C	−0.673630	3.730569	0.228842
C	−1.511380	0.114092	0.007300
C	−1.116915	−1.039014	−0.737138
C	−2.800492	−0.095685	0.602365
C	1.511317	0.114990	−0.007253
C	2.800519	−0.094004	−0.602398
C	3.340859	−1.309040	−0.345096
H	3.282964	0.627631	−1.244612
C	1.117544	−1.038398	0.737112
S	2.316684	−2.280441	0.691677
C	−3.340055	−1.311069	0.345073
S	−2.315266	−2.281816	−0.691704
H	−3.283389	0.625640	1.244587
H	−0.356614	−1.074058	−1.497480
H	−4.269088	−1.716206	0.712387
H	0.357269	−1.073925	1.497462
H	4.270149	−1.713586	−0.712409
H	2.385753	2.561632	−0.671155
H	1.182303	4.668562	−0.402743
H	−1.185253	4.667847	0.402492
H	−2.387315	2.560166	0.671233

C1–C1' = 2.58 Å

$E(S_1)$ = −1336.01696739 Ha

C	1.315872	0.720302	0.122831
C	1.313367	−0.724874	−0.122720
C	2.565564	1.346584	0.358720
C	2.560887	−1.355453	−0.358669
C	3.752414	0.673602	0.203572
C	3.750053	−0.686538	−0.203672
C	0.123748	−1.500453	0.011135
C	−1.039129	−1.064930	0.722979
C	−0.100710	−2.791332	−0.568964
C	0.128956	1.499986	−0.011011
C	−0.090919	2.791784	0.568784
C	−1.323279	3.304888	0.333292
H	0.635158	3.297036	1.188216
C	−1.035478	1.068360	−0.722664
S	−2.299739	2.249144	−0.665674
C	−1.334873	−3.300129	−0.333592
S	−2.307569	−2.241221	0.665690
H	0.623521	−3.299004	−1.188581
H	−1.055313	−0.326989	1.506162

H	−1.751853	−4.224329	−0.700079
H	−1.054324	0.330192	−1.505577
H	−1.736989	4.230648	0.699543
H	2.584293	2.403275	0.590339
H	4.690863	1.189359	0.357683
H	4.686705	−1.205536	−0.357838
H	2.576011	−2.412208	−0.590248

C1–C1' = 2.48 Å

$E(S_1)$ = −1336.01838916 Ha

C	−1.332383	0.724143	−0.104363
C	−1.332116	−0.724647	0.104273
C	−2.580857	1.360939	−0.316622
C	−2.580358	−1.361898	0.316546
C	−3.768734	0.685227	−0.180889
C	−3.768480	−0.686590	0.180943
C	−0.136412	−1.490234	−0.027921
C	1.037280	−1.018079	−0.705062
C	0.095830	−2.784947	0.534843
C	−0.136988	1.490193	0.027931
C	0.094738	2.785042	−0.534727
C	1.343716	3.269980	−0.321488
H	−0.634810	3.313893	−1.130110
C	1.036913	1.018403	0.704964
S	2.323194	2.182165	0.637897
C	1.344972	−3.269445	0.321578
S	2.324015	−2.181334	−0.637914
H	−0.633490	−3.314027	1.130303
H	1.041117	−0.305159	−1.512170
H	1.767899	−4.191911	0.685812
H	1.041052	0.305393	1.511995
H	1.766295	4.192620	−0.685685
H	−2.597188	2.424008	−0.517321
H	−4.706539	1.207045	−0.317766
H	−4.706092	−1.208731	0.317908
H	−2.596309	−2.424967	0.517266

C1–C1' = 2.38 Å

$E(S_1)$ = −1336.01986329 Ha

C	−1.347632	0.725849	−0.087116
C	−1.347706	−0.725737	0.087157
C	−2.595786	1.369641	−0.278228
C	−2.595926	−1.369413	0.278237
C	−3.783914	0.690656	−0.160418
C	−3.783984	−0.690305	0.160443

C	−0.145913	−1.480736	−0.043829
C	1.037259	−0.971418	−0.684776
C	0.094489	−2.779405	0.500957
C	−0.145758	1.480726	0.043809
C	0.094764	2.779343	−0.501048
C	1.357257	3.240624	−0.309110
H	−0.638832	3.329888	−1.071494
C	1.037358	0.971334	0.684804
S	2.341437	2.123335	0.608653
C	1.356962	−3.240760	0.309065
S	2.341256	−2.123512	−0.608622
H	−0.639160	−3.329921	1.071364
H	1.030512	−0.286775	−1.517187
H	1.785351	−4.162141	0.669843
H	1.030498	0.286765	1.517282
H	1.785734	4.161952	−0.669921
H	−2.611209	2.437624	−0.451262
H	−4.721614	1.216459	−0.281981
H	−4.721737	−1.216032	0.281925
H	−2.611461	−2.437399	0.451243

C1–C1' = 2.28 Å

$E(S_1) = -1336.02139461$ Ha

C	1.361492	−0.726816	−0.070374
C	1.361467	0.726867	0.070386
C	2.609583	−1.376741	−0.239654
C	2.609538	1.376831	0.239680
C	3.797732	−0.694423	−0.139642
C	3.797710	0.694566	0.139581
C	0.153798	1.471602	−0.058199
C	−1.038072	0.926025	−0.662354
C	−0.093741	2.774168	0.468382
C	0.153849	−1.471595	0.058232
C	−0.093595	−2.774225	−0.468238
C	−1.367797	−3.214527	−0.297954
H	0.644317	−3.344780	−1.013084
C	−1.038087	−0.926016	0.662252
S	−2.357316	−2.070621	0.577837
C	−1.367937	3.214449	0.298008
S	−2.357370	2.070546	−0.577884
H	0.644105	3.344701	1.013342
H	−1.024035	0.271724	−1.520205
H	−1.800589	4.135330	0.655206
H	−1.024158	−0.271581	1.520000
H	−1.800386	−4.135454	−0.655109
H	2.624477	−2.448918	−0.384578
H	4.735619	−1.223385	−0.245270

H	4.735581	1.223568	0.245159
H	2.624397	2.449003	0.384647

C1–C1' = 2.18 Å

$E(S_1) = -1336.02301205$ Ha

C	1.374066	−0.727623	−0.054303
C	1.373976	0.727746	0.054191
C	2.622051	−1.382599	−0.202334
C	2.621875	1.382863	0.202312
C	3.810387	−0.697546	−0.119342
C	3.810297	0.697950	0.119385
C	0.161020	1.462757	−0.072285
C	−1.038795	0.881586	−0.638508
C	−0.092972	2.769114	0.435982
C	0.161209	−1.462780	0.072297
C	−0.092662	−2.769180	−0.435918
C	−1.377151	−3.190319	−0.287797
H	0.649247	−3.358407	−0.955122
C	−1.038703	−0.881622	0.638340
S	−2.371825	−2.021641	0.545761
C	−1.377503	3.190136	0.287895
S	−2.372064	2.021416	−0.545736
H	0.648875	3.358375	0.955233
H	−1.020660	0.259677	−1.521720
H	−1.813504	4.110691	0.642091
H	−1.020680	−0.259478	1.521394
H	−1.813070	−4.110927	−0.641957
H	2.636612	−2.458180	−0.319549
H	4.748336	−1.229313	−0.209378
H	4.748178	1.229821	0.209520
H	2.636305	2.458437	0.319615

C1–C1' = 2.08 Å

$E(S_1) = -1336.02482953$ Ha

C	1.385653	−0.728500	−0.038191
C	1.385822	0.728277	0.038372
C	2.633278	−1.387884	−0.165553
C	2.633608	1.387383	0.165582
C	3.822162	−0.700708	−0.099530
C	3.822330	0.699929	0.099525
C	0.168119	1.454135	−0.086330
C	−1.039320	0.837871	−0.613349
C	−0.091261	2.764550	0.402400
C	0.167774	−1.454107	0.086327
C	−0.091895	−2.764334	−0.402756

C	−1.385708	−3.167593	−0.278387
H	0.653897	−3.371650	−0.895135
C	−1.039467	−0.837833	0.613787
S	−2.386215	−1.975395	0.510888
C	−1.385024	3.167992	0.278061
S	−2.385807	1.975779	−0.510841
H	0.654681	3.371861	0.894557
H	−1.020933	0.249665	−1.521163
H	−1.822845	4.088868	0.629411
H	−1.020832	−0.250167	1.521939
H	−1.823723	−4.088302	−0.629930
H	2.647431	−2.466138	−0.255260
H	4.759970	−1.235061	−0.174605
H	4.760265	1.234071	0.174506
H	2.648017	2.465643	0.255177

C1–C1' = 1.98 Å

$E(S_1)$ = −1336.02714541 Ha

C	1.397010	−0.728727	−0.022661
C	1.396506	0.729157	0.022657
C	2.644626	−1.390923	−0.128398
C	2.643710	1.392137	0.128320
C	3.833981	−0.701223	−0.079589
C	3.833519	0.703220	0.079420
C	0.173997	1.445730	−0.099544
C	−1.040897	0.794934	−0.587246
C	−0.090008	2.760377	0.367681
C	0.174946	−1.445940	0.099673
C	−0.088573	−2.760747	−0.367362
C	−1.390533	−3.148602	−0.268356
H	0.661651	−3.385823	−0.830231
C	−1.040142	−0.795430	0.587144
S	−2.399799	−1.934130	0.471445
C	−1.392083	3.147835	0.268700
S	−2.400858	1.933300	−0.471628
H	0.659989	3.385631	0.830676
H	−1.024899	0.244996	−1.520710
H	−1.829868	4.070112	0.616650
H	−1.024431	−0.245191	1.520441
H	−1.828014	−4.071085	−0.616138
H	2.659228	−2.471171	−0.189781
H	4.771853	−1.237429	−0.139156
H	4.771039	1.240050	0.138885
H	2.657610	2.472398	0.189670

C1–C1' = 1.88 Å

$E(S_1) = -1336.03012665$ Ha

C	1.407387	−0.729126	−0.008680
C	1.406751	0.729577	0.008785
C	2.654722	−1.393524	−0.093724
C	2.653546	1.395005	0.093591
C	3.845126	−0.702053	−0.060862
C	3.844545	0.704558	0.060480
C	0.180120	1.437891	−0.110182
C	−1.042720	0.753829	−0.558498
C	−0.086620	2.758012	0.330494
C	0.181347	−1.438256	0.110624
C	−0.084873	−2.758484	−0.330063
C	−1.393751	−3.133384	−0.256461
H	0.669938	−3.401553	−0.759957
C	−1.041730	−0.754514	0.558621
S	−2.414571	−1.897425	0.427046
C	−1.395622	3.132485	0.256707
S	−2.415858	1.896471	−0.427360
H	0.667909	3.401369	0.760443
H	−1.032047	0.247624	−1.518452
H	−1.830419	4.059261	0.596616
H	−1.031497	−0.248064	1.518449
H	−1.828266	−4.060259	−0.596448
H	2.669683	−2.474959	−0.128156
H	4.782829	−1.239985	−0.105746
H	4.781800	1.243280	0.105183
H	2.667582	2.476452	0.128089

C1–C1' = 1.78 Å

$E(S_1) = -1336.03386182$ Ha

C	1.416685	0.729732	−0.002262
C	1.416984	−0.729580	0.001739
C	2.663303	1.395990	0.064501
C	2.663630	−1.395758	−0.064353
C	3.855435	0.703729	0.044922
C	3.855694	−0.703396	−0.044369
C	0.187117	−1.430775	0.116975
C	−1.043242	−0.715024	0.527307
C	−0.081440	−2.757840	−0.289639
C	0.186774	1.430656	−0.117978
C	−0.081588	2.757872	0.288838
C	−1.396585	3.121142	0.240588
H	0.677148	3.420258	0.681186
C	−1.043582	0.714552	−0.528141
S	−2.430663	1.863069	−0.377082
C	−1.396357	−3.121453	−0.238662

S	−2.430661	−1.862936	0.377055
H	0.677009	−3.420526	−0.682009
H	−1.039494	−0.257696	1.513595
H	−1.826423	−4.055982	−0.563353
H	−1.039968	0.257023	−1.514326
H	−1.826701	4.054943	0.567240
H	2.678338	2.477881	0.076351
H	4.792594	1.243506	0.077186
H	4.792982	−1.242959	−0.076192
H	2.678745	−2.477652	−0.076031

C1–C1' = 1.68 Å

$E(S_1)$ = −1336.03725512 Ha

C	1.424077	−0.729301	0.006587
C	1.424097	0.729241	−0.006531
C	2.671268	−1.397507	−0.051632
C	2.671295	1.397430	0.051686
C	3.863330	−0.703620	−0.037042
C	3.863349	0.703527	0.037102
C	0.192932	1.421340	−0.119507
C	−1.046007	0.677224	−0.494322
C	−0.076586	2.761716	0.242764
C	0.192899	−1.421367	0.119514
C	−0.076703	−2.761656	−0.243041
C	−1.392959	−3.116178	−0.213471
H	0.686269	−3.444221	−0.590177
C	−1.045997	−0.677246	0.494451
S	−2.445119	−1.832737	0.322627
C	−1.392837	3.116279	0.213190
S	−2.445088	1.832776	−0.322486
H	0.686417	3.444335	0.589715
H	−1.051726	0.267766	−1.503405
H	−1.817153	4.063011	0.509846
H	−1.051641	−0.267814	1.503542
H	−1.817357	−4.062809	−0.510325
H	2.686907	−2.479269	−0.053549
H	4.800835	−1.243174	−0.062438
H	4.800866	1.243061	0.062451
H	2.686947	2.479192	0.053595

C1–C1' = 1.58 Å

$E(S_1)$ = −1336.03937078 Ha

C	1.432358	−0.729201	0.010172
C	1.432357	0.729210	−0.010170
C	2.679077	−1.398182	−0.038160

C	2.679073	1.398196	0.038138
C	3.872940	−0.702804	−0.029206
C	3.872940	0.702822	0.029177
C	0.198188	1.413829	−0.117401
C	−1.046332	0.642613	−0.456912
C	−0.072305	2.765011	0.189576
C	0.198194	−1.413827	0.117432
C	−0.072282	−2.765027	−0.189487
C	−1.392334	−3.111508	−0.180772
H	0.694041	−3.469884	−0.480175
C	−1.046340	−0.642613	0.456903
S	−2.459586	−1.804537	0.254083
C	−1.392356	3.111494	0.180806
S	−2.459588	1.804533	−0.254126
H	0.694006	3.469860	0.480318
H	−1.065624	0.291101	−1.489522
H	−1.810211	4.071535	0.442756
H	−1.065668	−0.291094	1.489511
H	−1.810178	−4.071558	−0.442708
H	2.695573	−2.479814	−0.032344
H	4.810111	−1.243184	−0.048820
H	4.810109	1.243204	0.048774
H	2.695563	2.479828	0.032314

Cartesian coordinates (in Å) and electronic energies (E, in Ha) of S_0 geometries of 2

Geometries optimized at the B3LYP/cc-pVTZ/SMD level of theory.

2o

$E(S_0) = -1258.5696407$ Ha (all vibrational frequencies real)

C	−0.682705	1.222024	0.016029
C	0.682720	1.221999	−0.016485
C	−0.664045	2.781447	0.023815
C	0.664102	2.781436	−0.024253
H	−1.429411	3.542148	0.050405
H	1.429475	3.542127	−0.050890
C	1.800256	0.326617	−0.010585
C	1.796927	−1.045242	0.168214
C	3.145814	0.817670	−0.177932
C	−1.800255	0.326654	0.010389
C	−3.145716	0.817643	0.178695
C	−4.091867	−0.153936	0.130390
H	−3.376801	1.861227	0.333442
C	−1.797022	−1.045131	−0.168945
S	−3.381935	−1.708954	−0.130731
C	4.091944	−0.153918	−0.129409
S	3.381866	−1.709037	0.130705
H	3.376991	1.861311	−0.332147
H	0.961975	−1.696231	0.354638
H	5.160217	−0.055114	−0.232894
H	−0.962188	−1.696022	−0.356251
H	−5.160077	−0.055188	0.234579

2c

$E(S_0) = -1258.5850988$ Ha (all vibrational frequencies real)

C	0.738760	1.802602	0.016840
C	−0.738759	1.802602	−0.015977
C	0.683742	3.274153	−0.004242
C	−0.683703	3.274175	0.003995
H	1.429772	4.054390	−0.004592
H	−1.429706	4.054436	0.003693
C	−1.470548	0.686394	−0.133483
C	−0.674558	−0.606543	−0.356215
C	−2.890072	0.432606	−0.085799
C	1.470583	0.686360	0.133700
C	2.890085	0.432579	0.085375
C	3.207327	−0.865159	−0.054038
H	3.628960	1.221723	0.090435
C	0.674604	−0.606591	0.356543

S	1.838498	−1.976327	−0.119303
C	−3.207357	−0.865139	0.053500
S	−1.838543	−1.976293	0.119368
H	−3.628955	1.221740	−0.091144
H	0.511042	−0.725835	1.430427
H	−0.510955	−0.725693	−1.430111
H	4.201372	−1.273541	−0.160032
H	−4.201442	−1.273526	0.159100

2ts

$E(S_0)$ = −1258.5326445 Ha (one imaginary vibrational frequency)

C	−0.778956	1.788385	0.008775
C	0.778882	1.788442	−0.009505
C	−0.728780	3.170465	0.028785
C	0.728611	3.170527	−0.028163
H	−1.474929	3.951029	0.000782
H	1.474709	3.951124	0.000311
C	1.552054	0.646627	0.097234
C	0.917738	−0.566959	0.570147
C	2.887682	0.408113	−0.382499
C	−1.552052	0.646553	−0.097578
C	−2.887680	0.408112	0.382232
C	−3.266064	−0.885938	0.311409
H	−3.494234	1.180218	0.834008
C	−0.917732	−0.567113	−0.570301
S	−2.056925	−1.911596	−0.430552
C	3.266119	−0.885920	−0.311351
S	2.057005	−1.911429	0.430816
H	3.494214	1.180127	−0.834461
H	−0.315875	−0.585361	−1.462378
H	0.315814	−0.585014	1.462183
H	−4.176135	−1.325736	0.688254
H	4.176231	−1.325767	−0.688038

Cartesian coordinates (in Å) and electronic energies (E, in Ha) of S_1 geometries of 2

Geometry optimized at the TD-B3LYP/cc-pVTZ/SMD level of theory.

2o (S_1)

$E(S_1)$ = −1258.5435996 Ha (all vibrational frequencies real)

C	0.733467	1.288902	−0.037143
C	−0.733456	1.288901	0.037139
C	0.701792	2.732891	−0.048795
C	−0.701771	2.732889	0.048720
H	1.459600	3.498403	−0.096518
H	−1.459580	3.498400	0.096466
C	−1.814649	0.372290	0.020392
C	−1.763001	−0.996223	−0.217579
C	−3.179048	0.802218	0.232047
C	1.814654	0.372289	−0.020365
C	3.179060	0.802217	−0.231978
C	4.083509	−0.203174	−0.161594
H	3.447796	1.827695	−0.439658
C	1.762990	−0.996231	0.217554
S	3.318673	−1.722215	0.177626
C	−4.083504	−0.203168	0.161665
S	−3.318691	−1.722197	−0.177653
H	−3.447773	1.827693	0.439750
H	−0.904259	−1.597788	−0.458357
H	−5.152179	−0.154397	0.294145
H	0.904236	−1.597807	0.458256
H	5.152188	−0.154404	−0.294047

Geometries optimized at the TD-B3LYP/cc-pVTZ/SMD level of theory with all geometric degrees of freedom relaxed except the C1–C1' distance.

C1–C1' = 3.51 Å

$E(S_1)$ = −1258.52007599 Ha

C	−0.732472	1.305553	0.040190
C	0.732502	1.305537	−0.040791
C	−0.701670	2.749586	0.053327
C	0.701785	2.749581	−0.053046
H	−1.459273	3.515016	0.105736
H	1.459448	3.514993	−0.104848
C	1.805666	0.380395	−0.021662
C	1.738399	−0.983467	0.239243
C	3.172585	0.790220	−0.253249
C	−1.805663	0.380444	0.021202
C	−3.172496	0.790285	0.253284

C	−4.065050	−0.225337	0.176377
H	−3.451160	1.808961	0.480710
C	−1.738503	−0.983430	−0.239668
S	−3.285061	−1.728990	−0.195382
C	4.065114	−0.225392	−0.175895
S	3.284969	−1.729041	0.195556
H	3.451333	1.808880	−0.480646
H	0.876279	−1.569146	0.505267
H	5.132719	−0.192470	−0.321187
H	−0.876455	−1.569176	−0.505781
H	−5.132597	−0.192405	0.322096

C1–C1' = 3.41 Å

$E(S_1) = -1258.51971429$ Ha

C	−0.730490	1.342639	0.045285
C	0.730451	1.342641	−0.045059
C	−0.701832	2.786924	0.059220
C	0.701814	2.786923	−0.059046
H	−1.459216	3.552250	0.116753
H	1.459209	3.552236	−0.116607
C	1.785651	0.398488	−0.023146
C	1.682750	−0.956086	0.274113
C	3.158107	0.764539	−0.287497
C	−1.785673	0.398479	0.023224
C	−3.158215	0.764562	0.287099
C	−4.023318	−0.273847	0.198168
H	−3.460264	1.768866	0.546641
C	−1.682674	−0.956145	−0.273774
S	−3.208430	−1.744657	−0.225998
C	4.023264	−0.273824	−0.198547
S	3.208500	−1.744590	0.226018
H	3.460056	1.768786	−0.547374
H	0.813898	−1.507996	0.584547
H	5.088352	−0.274913	−0.364460
H	−0.813717	−1.508111	−0.583821
H	−5.088455	−0.274922	0.363762

C1–C1' = 3.31 Å

$E(S_1) = -1258.51899900$ Ha

C	−0.728506	1.379598	0.050155
C	0.728548	1.379617	−0.050064
C	−0.701633	2.824258	0.065373
C	0.701725	2.824281	−0.065322
H	−1.458612	3.589533	0.129100
H	1.458723	3.589548	−0.128853

C	1.765576	0.416815	−0.024479
C	1.626330	−0.927105	0.306145
C	3.142729	0.737438	−0.317428
C	−1.765548	0.416811	0.024406
C	−3.142716	0.737505	0.317229
C	−3.978804	−0.323723	0.217128
H	−3.468752	1.726159	0.606368
C	−1.626313	−0.927184	−0.305920
S	−3.128876	−1.759755	−0.254860
C	3.978785	−0.323804	−0.217234
S	3.128801	−1.759781	0.254847
H	3.468772	1.726048	−0.606708
H	0.750411	−1.442724	0.654805
H	5.040257	−0.360121	−0.401284
H	−0.750363	−1.442937	−0.654302
H	−5.040285	−0.359973	0.401140

C1–C1' = 3.21 Å

$E(S_1)$ = −1258.51783529 Ha

C	−0.726599	1.416821	0.053586
C	0.726708	1.416789	−0.054308
C	−0.701370	2.861902	0.070452
C	0.701675	2.861890	−0.070306
H	−1.457933	3.627200	0.139046
H	1.458349	3.627142	−0.138103
C	1.744786	0.434961	−0.024558
C	1.568665	−0.896837	0.338033
C	3.124958	0.708447	−0.345554
C	−1.744754	0.435067	0.024059
C	−3.124726	0.708574	0.345905
C	−3.930272	−0.375357	0.235740
H	−3.474328	1.680143	0.664024
C	−1.568905	−0.896710	−0.338740
S	−3.046215	−1.773933	−0.283663
C	3.930401	−0.375513	−0.234946
S	3.045965	−1.774101	0.283799
H	3.474781	1.680028	−0.663392
H	0.687508	−1.374584	0.724026
H	4.986586	−0.448666	−0.437633
H	−0.688031	−1.374430	−0.725415
H	−4.986331	−0.448486	0.439095

C1–C1' = 3.11 Å

$E(S_1)$ = −1258.51617467 Ha

C	−0.724844	1.452214	0.056251

C	0.724932	1.452187	−0.056590
C	−0.701348	2.897870	0.073721
C	0.701603	2.897856	−0.073671
H	−1.457664	3.663149	0.145381
H	1.457983	3.663088	−0.145079
C	1.724079	0.452055	−0.023104
C	1.511623	−0.868301	0.363557
C	3.106460	0.680783	−0.365798
C	−1.724052	0.452140	0.022826
C	−3.106298	0.680880	0.366064
C	−3.881473	−0.424487	0.248121
H	−3.478915	1.636332	0.706153
C	−1.511794	−0.868191	−0.364023
S	−2.964036	−1.787153	−0.307011
C	3.881555	−0.424611	−0.247616
S	2.963846	−1.787282	0.307068
H	3.479226	1.636238	−0.705711
H	0.627646	−1.312255	0.780773
H	4.931750	−0.533008	−0.465335
H	−0.628046	−1.312093	−0.781781
H	−4.931587	−0.532857	0.466247

C1–C1' = 3.01 Å

$E(S_1) = -1258.51389677$ Ha

C	−0.723308	1.486506	0.056878
C	0.723256	1.486548	−0.056188
C	−0.701624	2.932793	0.074300
C	0.701534	2.932826	−0.074297
H	−1.457974	3.698110	0.145425
H	1.457814	3.698146	−0.146038
C	1.703211	0.468609	−0.019287
C	1.455003	−0.841007	0.384953
C	3.086767	0.654260	−0.379856
C	−1.703219	0.468529	0.019569
C	−3.086995	0.654246	0.379277
C	−3.831551	−0.471697	0.256387
H	−3.482300	1.594541	0.735672
C	−1.454780	−0.841183	−0.384226
S	−2.881482	−1.800252	−0.325583
C	3.831371	−0.471680	−0.257242
S	2.881634	−1.800108	0.325586
H	3.481858	1.594480	−0.736682
H	0.569815	−1.252328	0.830220
H	4.874833	−0.613545	−0.487780
H	−0.569306	−1.252660	−0.828767
H	−4.875163	−0.613487	0.486294

C1–C1' = 2.91 Å

$E(S_1) = -1258.51093650$ Ha

C	−0.721620	1.522346	0.056193
C	0.721628	1.522342	−0.056104
C	−0.701530	2.969870	0.074459
C	0.701576	2.969864	−0.074567
H	−1.457851	3.735235	0.145628
H	1.457918	3.735204	−0.145741
C	1.681446	0.485935	−0.013937
C	1.395573	−0.810212	0.411170
C	3.064149	0.624105	−0.395390
C	−1.681439	0.485937	0.014034
C	−3.064160	0.624104	0.395427
C	−3.775014	−0.523205	0.267888
H	−3.482476	1.546369	0.772201
C	−1.395560	−0.810205	−0.411088
S	−2.792784	−1.813062	−0.347146
C	3.774993	−0.523218	−0.267931
S	2.792767	−1.813086	0.347095
H	3.482453	1.546379	−0.772154
H	0.510899	−1.184305	0.887748
H	4.809445	−0.701107	−0.513607
H	−0.510886	−1.184277	−0.887682
H	−4.809482	−0.701085	0.513503

C1–C1' = 2.81 Å

$E(S_1) = -1258.50721234$ Ha

C	−0.720016	1.558712	0.054403
C	0.720031	1.558720	−0.054014
C	−0.701421	3.007787	0.072689
C	0.701474	3.007788	−0.072958
H	−1.457959	3.773225	0.141064
H	1.457989	3.773204	−0.141749
C	1.659057	0.503927	−0.005965
C	1.334442	−0.777290	0.439472
C	3.038429	0.591791	−0.410250
C	−1.659030	0.503901	0.006243
C	−3.038479	0.591769	0.410271
C	−3.712763	−0.577403	0.279500
H	−3.480179	1.494458	0.807319
C	−1.334366	−0.777304	−0.439189
S	−2.698547	−1.825415	−0.369410
C	3.712681	−0.577425	−0.279746
S	2.698540	−1.825444	0.369291
H	3.480076	1.494501	−0.807307

H	0.453844	−1.111793	0.950268
H	4.735364	−0.793989	−0.543065
H	−0.453732	−1.111768	−0.949950
H	−4.735515	−0.793934	0.542576

C1–C1' = 2.71 Å

$E(S_1) = -1258.50266979$ Ha

C	−0.718536	1.595657	0.049976
C	0.718282	1.595682	−0.050090
C	−0.701429	3.047032	0.069550
C	0.700770	3.047039	−0.069661
H	−1.458304	3.812491	0.133991
H	1.457533	3.812638	−0.134084
C	1.635773	0.522523	0.005867
C	1.271049	−0.743042	0.469330
C	3.009313	0.558098	−0.422127
C	−1.635877	0.522390	−0.005713
C	−3.009389	0.557737	0.422352
C	−3.644291	−0.633640	0.291252
H	−3.474525	1.439561	0.839129
C	−1.270922	−0.743067	−0.469298
S	−2.598812	−1.838019	−0.390544
C	3.644432	−0.633185	−0.291123
S	2.599154	−1.837856	0.390401
H	3.474335	1.440038	−0.838795
H	0.398383	−1.035508	1.016982
H	4.652739	−0.889292	−0.573420
H	−0.398156	−1.035295	−1.016915
H	−4.652547	−0.889979	0.573513

C1–C1' = 2.61 Å

$E(S_1) = -1258.49737050$ Ha

C	−0.717402	1.634380	0.042058
C	0.716154	1.634541	−0.042786
C	−0.701958	3.089092	0.063002
C	0.698989	3.089201	−0.062116
H	−1.459522	3.854493	0.119607
H	1.455956	3.855248	−0.119345
C	1.611021	0.543068	0.023534
C	1.203634	−0.705484	0.504371
C	2.974833	0.521832	−0.431575
C	−1.611645	0.542527	−0.024086
C	−2.975293	0.520376	0.431399
C	−3.565054	−0.694964	0.304892
H	−3.465128	1.379076	0.867752

C	−1.203125	−0.705765	−0.504689
S	−2.487906	−1.851757	−0.411013
C	3.565778	−0.692925	−0.304428
S	2.489581	−1.850649	0.411105
H	3.464090	1.380803	−0.868074
H	0.346901	−0.950877	1.097615
H	4.555917	−0.990946	−0.608832
H	−0.345822	−0.950624	−1.097306
H	−4.554786	−0.993955	0.609653

C1–C1' = 2.51 Å

$E(S_1) = -1258.49158209$ Ha

C	−0.715015	1.674810	0.031785
C	0.714848	1.674796	−0.032750
C	−0.698969	3.134768	0.053644
C	0.698632	3.134771	−0.053098
H	−1.456640	3.900849	0.100389
H	1.456308	3.900937	−0.098695
C	1.585701	0.565532	0.046996
C	1.130152	−0.664587	0.544875
C	2.934386	0.480515	−0.439224
C	−1.585829	0.565532	−0.047572
C	−2.934237	0.480396	0.439390
C	−3.472864	−0.760029	0.320360
H	−3.449268	1.312569	0.897671
C	−1.130325	−0.664569	−0.545532
S	−2.365761	−1.865216	−0.432841
C	3.473176	−0.759805	−0.319704
S	2.365877	−1.865090	0.433001
H	3.449537	1.312691	−0.897368
H	0.304097	−0.852426	1.199324
H	4.439041	−1.104860	−0.651202
H	−0.304379	−0.852415	−1.200121
H	−4.438482	−1.105220	0.652428

C1–C1' = 2.41 Å

$E(S_1) = -1258.48586437$ Ha

C	−0.713383	1.706814	0.027432
C	0.713365	1.706813	−0.027481
C	−0.695910	3.172228	0.051314
C	0.695888	3.172226	−0.051293
H	−1.452614	3.939722	0.093293
H	1.452596	3.939720	−0.093258
C	1.561960	0.588396	0.067507
C	1.058121	−0.630505	0.576244

C	2.897075	0.447146	−0.438630
C	−1.561981	0.588404	−0.067550
C	−2.897063	0.447127	0.438670
C	−3.386264	−0.814614	0.329303
H	−3.436037	1.255165	0.912603
C	−1.058128	−0.630498	−0.576271
S	−2.251837	−1.878577	−0.450113
C	3.386290	−0.814590	−0.329250
S	2.251843	−1.878575	0.450101
H	3.436069	1.255196	−0.912517
H	0.286733	−0.756112	1.309571
H	4.328552	−1.198396	−0.685215
H	−0.286725	−0.756108	−1.309583
H	−4.328493	−1.198441	0.685331

C1–C1' = 2.31 Å

$E(S_1)$ = −1258.48122089 Ha

C	−0.709865	1.728774	0.020386
C	0.709754	1.728781	−0.020348
C	−0.689828	3.207405	0.045882
C	0.689722	3.207413	−0.045887
H	−1.446249	3.975625	0.083055
H	1.446139	3.975632	−0.083131
C	1.543508	0.605810	0.086559
C	0.994057	−0.612613	0.588217
C	2.870734	0.428366	−0.420519
C	−1.543579	0.605785	−0.086627
C	−2.870850	0.428301	0.420321
C	−3.322045	−0.849697	0.328154
H	−3.432126	1.222610	0.891820
C	−0.993872	−0.612720	−0.587789
S	−2.162994	−1.893764	−0.447758
C	3.322047	−0.849581	−0.328231
S	2.163048	−1.893706	0.447685
H	3.431921	1.222671	−0.892128
H	0.291401	−0.690069	1.397026
H	4.249046	−1.256303	−0.698710
H	−0.290654	−0.690343	−1.396104
H	−4.249030	−1.256457	0.698629

C1–C1' = 2.21 Å

$E(S_1)$ = −1258.47808111 Ha

C	−0.706723	1.744682	0.011231
C	0.706708	1.744669	−0.011292
C	−0.684674	3.235689	0.037610

C	0.684688	3.235678	−0.037599
H	−1.440894	4.004639	0.066948
H	1.440923	4.004615	−0.066881
C	1.530335	0.619165	0.103483
C	0.938240	−0.604892	0.583398
C	2.855768	0.420209	−0.390451
C	−1.530366	0.619188	−0.103513
C	−2.855752	0.420195	0.390532
C	−3.279123	−0.869800	0.319098
H	−3.438707	1.208644	0.845578
C	−0.938278	−0.604852	−0.583472
S	−2.095354	−1.908980	−0.430022
C	3.279151	−0.869781	−0.319034
S	2.095356	−1.908993	0.430006
H	3.438753	1.208685	−0.845412
H	0.299985	−0.652609	1.449534
H	4.199899	−1.287347	−0.693201
H	−0.300005	−0.652571	−1.449597
H	−4.199832	−1.287393	0.693333

C1–C1' = 2.11 Å

$E(S_1)$ = −1258.47632015 Ha

C	−0.704780	1.759385	0.001181
C	0.704614	1.759423	−0.001191
C	−0.681916	3.258666	0.027133
C	0.681672	3.258704	−0.027019
H	−1.438129	4.028119	0.045041
H	1.437844	4.028200	−0.044815
C	1.519027	0.629817	0.118433
C	0.886986	−0.599108	0.571191
C	2.844601	0.414537	−0.358123
C	−1.519115	0.629734	−0.118513
C	−2.844739	0.414373	0.357866
C	−3.245288	−0.885316	0.306998
H	−3.448754	1.199539	0.790838
C	−0.886850	−0.599195	−0.570942
S	−2.036738	−1.922228	−0.406919
C	3.245261	−0.885114	−0.307250
S	2.036941	−1.922061	0.407012
H	3.448476	1.199721	−0.791257
H	0.301639	−0.631405	1.477487
H	4.163727	−1.309575	−0.679349
H	−0.301116	−0.631586	−1.476988
H	−4.163782	−1.309825	0.678974

C1–C1' = 2.01 Å

$E(S_1) = -1258.47572145$ Ha

C	0.703664	1.774218	0.009589
C	−0.703544	1.774264	−0.009388
C	0.680416	3.278159	−0.015253
C	−0.680172	3.278216	0.015039
H	1.436517	4.048002	−0.019510
H	−1.436214	4.048116	0.019138
C	−1.508312	0.639120	−0.132393
C	−0.837270	−0.593137	−0.555018
C	−2.834136	0.408671	0.326260
C	1.508353	0.639026	0.132487
C	2.834189	0.408537	−0.326105
C	3.214680	−0.899364	−0.294393
H	3.458276	1.190566	−0.735986
C	0.837218	−0.593207	0.555015
S	1.982026	−1.933653	0.382233
C	−3.214717	−0.899200	0.294476
S	−1.982186	−1.933508	−0.382353
H	−3.458134	1.190705	0.736267
H	0.296527	−0.618126	1.491279
H	−0.296615	−0.618005	−1.491305
H	4.132800	−1.329341	−0.660941
H	−4.132825	−1.329151	0.661082

C1–C1' = 1.91 Å

$E(S_1) = -1258.47611565$ Ha

C	0.702558	1.788572	0.015950
C	−0.703491	1.788178	−0.021467
C	0.678309	3.295050	−0.002425
C	−0.680642	3.294433	0.006169
H	1.433761	4.065497	0.008553
H	−1.436793	4.064265	−0.000197
C	−1.498596	0.647106	−0.144709
C	−0.790023	−0.587860	−0.536412
C	−2.825005	0.403093	0.294438
C	1.498305	0.647992	0.142098
C	2.825441	0.404026	−0.294945
C	3.189087	−0.910691	−0.279821
H	3.468591	1.183138	−0.680508
C	0.790038	−0.586823	0.534889
S	1.933558	−1.943607	0.356663
C	−3.187918	−0.911855	0.281465
S	−1.932725	−1.944814	−0.355410
H	−3.468203	1.182394	0.679526
H	0.291427	−0.607464	1.496428
H	−0.292219	−0.609582	−1.498296

H	4.109017	−1.344832	−0.636804
H	−4.107289	−1.346004	0.639861

C1–C1' = 1.81 Å

E(S_1) = −1258.47731879 Ha

C	0.702568	1.801835	0.025650
C	−0.703092	1.801607	−0.027593
C	0.678383	3.309154	0.005938
C	−0.679715	3.308853	−0.004448
H	1.433531	4.079729	0.025741
H	−1.435212	4.079104	−0.022647
C	−1.489645	0.655393	−0.151928
C	−0.745457	−0.582522	−0.512429
C	−2.819200	0.400503	0.258736
C	1.489493	0.655904	0.150880
C	2.819274	0.401150	−0.259102
C	3.168475	−0.919773	−0.262599
H	3.481910	1.178523	−0.614281
C	0.745578	−0.582040	0.511922
S	1.889892	−1.955083	0.322491
C	−3.167994	−0.920526	0.262987
S	−1.889325	−1.955747	−0.321915
H	−3.481974	1.177885	0.613629
H	0.292014	−0.603337	1.497445
H	−0.292033	−0.604291	−1.497997
H	4.093258	−1.355340	−0.605053
H	−4.092572	−1.356214	0.605851

C1–C1' = 1.71 Å

E(S_1) = −1258.47892764 Ha

C	−0.702850	1.814303	−0.030880
C	0.703205	1.814133	0.032203
C	−0.678438	3.321374	−0.012260
C	0.679325	3.321180	0.011276
H	−1.433236	4.092029	−0.039194
H	1.434368	4.091633	0.037083
C	1.481665	0.663318	0.154502
C	0.704637	−0.577999	0.483565
C	2.817208	0.400345	−0.215976
C	−1.481553	0.663650	−0.153710
C	−2.817281	0.400794	0.216146
C	−3.155150	−0.925671	0.237180
H	−3.500940	1.177426	0.531099
C	−0.704701	−0.577685	−0.483103
S	−1.851140	−1.967261	−0.276157

C	3.154765	−0.926183	−0.237698
S	1.850794	−1.967688	0.275842
H	3.500912	1.176965	−0.530857
H	−0.301364	−0.603146	−1.491885
H	0.301388	−0.603825	1.492366
H	−4.089559	−1.360340	0.553844
H	4.088968	−1.360912	−0.554884

C1–C1' = 1.61 Å

$E(S_1) = -1258.47975177$ Ha

C	−0.703529	1.825927	−0.031003
C	0.704113	1.825653	0.031980
C	−0.678364	3.331801	−0.013842
C	0.679784	3.331528	0.013056
H	−1.432795	4.102729	−0.041682
H	1.434585	4.102105	0.040374
C	1.475617	0.671189	0.147234
C	0.668081	−0.572507	0.448229
C	2.819202	0.400570	−0.167752
C	−1.475453	0.671724	−0.146697
C	−2.819191	0.401416	0.167886
C	−3.147886	−0.930682	0.202303
H	−3.525999	1.176196	0.432482
C	−0.668266	−0.572163	−0.447906
S	−1.816677	−1.979021	−0.217919
C	3.147410	−0.931632	−0.202617
S	1.816027	−1.979633	0.217667
H	3.526216	1.175195	−0.432237
H	−0.322257	−0.602535	−1.479369
H	0.322150	−0.603100	1.479705
H	−4.095610	−1.363946	0.479168
H	4.094988	−1.365117	−0.479643

Cartesian coordinates (in Å) and electronic energies (E, in Ha) of S_0 geometries of 3

Geometries optimized at the B3LYP/cc-pVTZ/SMD level of theory.

3o

$E(S_0) = -1338.5411761$ Ha (all vibrational frequencies real)

C	0.604990	1.206181	−0.035899
C	−0.737582	1.131195	0.035720
C	1.332906	2.534730	−0.125053
C	−1.608429	2.370852	0.123796
C	0.423232	3.722600	−0.428390
C	−0.836251	3.652374	0.427429
C	−1.494851	−0.144818	−0.004579
C	−1.412353	−1.066422	−1.013513
C	−2.456719	−0.538990	0.984541
C	1.500207	0.022750	0.004593
C	2.494326	−0.265472	−0.989072
C	3.219953	−1.385539	−0.723405
H	2.642986	0.335354	−1.874896
C	1.527773	−0.897255	1.017897
S	2.727010	−2.114205	0.764267
C	−3.050128	−1.734612	0.719905
S	−2.468810	−2.409358	−0.761219
H	−2.677964	0.044954	1.866483
H	−0.793997	−1.029835	−1.895176
H	−3.788193	−2.260151	1.303812
H	0.914113	−0.926386	1.903146
H	4.008605	−1.827468	−1.310480
H	1.865094	2.702041	0.819024
H	0.963732	4.655285	−0.255902
H	−1.476980	4.519283	0.255019
H	−2.155050	2.478022	−0.820767
H	2.113420	2.456050	−0.886013
H	0.141579	3.708384	−1.485808
H	−0.554856	3.669318	1.484871
H	−2.376131	2.206712	0.884082

3c

$E(S_0) = -1338.5286148$ Ha (all vibrational frequencies real)

C	−1.274182	0.729693	−0.019799
C	−1.274180	−0.729698	0.019791
C	−2.580445	1.494654	−0.138581
C	−2.580442	−1.494660	0.138570
C	−3.789055	0.613555	−0.452155
C	−3.789046	−0.613563	0.452171

H	−4.703438	1.195866	−0.324678
H	−4.703432	−1.195874	0.324713
H	−2.767978	−2.013869	−0.807531
H	−2.767971	2.013883	0.807511
C	−0.095504	−1.401669	−0.105422
C	1.194878	−0.645086	−0.383767
C	0.137717	−2.822167	−0.060853
C	−0.095508	1.401668	0.105418
C	0.137708	2.822167	0.060850
C	1.436171	3.175720	0.013982
H	−0.658000	3.552520	0.033455
C	1.194875	0.645089	0.383767
S	2.568740	1.828788	−0.001808
C	1.436180	−3.175717	−0.013981
S	2.568745	−1.828781	0.001812
H	−0.657989	−3.552524	−0.033462
H	1.263297	0.434114	1.459058
H	1.263302	−0.434112	−1.459058
H	1.827379	4.180913	−0.033802
H	1.827391	−4.180908	0.033803
H	−2.478237	−2.277029	0.893272
H	−3.760410	−0.291518	1.498039
H	−3.760442	0.291510	−1.498023
H	−2.478245	2.277008	−0.893300

3ts

$E(S_0) = -1338.4698176$ Ha (one imaginary vibrational frequency)

C	−1.314028	0.680878	0.061511
C	−1.314059	−0.680747	−0.061577
C	−2.595089	1.482984	0.094702
C	−2.595202	−1.482836	−0.094685
C	−3.827497	0.686845	−0.330919
C	−3.827597	−0.686628	0.331085
H	−4.731125	1.243766	−0.075967
H	−4.731289	−1.243397	0.076013
H	−2.738962	−1.872784	−1.109624
H	−2.738687	1.872994	1.109647
C	−0.049869	−1.415513	−0.167371
C	1.157674	−0.744879	−0.609898
C	0.218064	−2.678938	0.387436
C	−0.049781	1.415574	0.167247
C	0.218351	2.678852	−0.387777
C	1.536691	3.063968	−0.334841
H	−0.532952	3.290354	−0.866667
C	1.157636	0.744891	0.610021
S	2.525255	1.878123	0.470320
C	1.536371	−3.064167	0.334636

S	2.525176	−1.878255	−0.470129
H	−0.533361	−3.290455	0.866113
H	1.177286	0.172301	1.528150
H	1.177507	−0.172262	−1.528008
H	1.981239	3.956459	−0.744068
H	1.980775	−3.956756	0.743805
H	−2.487135	2.364943	−0.540931
H	−3.826455	0.562204	−1.418227
H	−3.826586	−0.562136	1.418404
H	−2.487142	−2.364820	0.540886

Cartesian coordinates (in Å) and electronic energies (E, in Ha) of S_1 geometries of 3

Geometries optimized at the TD-B3LYP/cc-pVTZ/SMD level of theory with all geometric degrees of freedom relaxed except the C1–C1' distance.

C1–C1' = 3.48 Å

$E(S_1)$ = −1338.41432837 Ha

C	0.655992	−0.954415	0.294653
C	−0.655753	−0.954592	−0.294480
C	1.094126	−2.211576	1.024595
C	−1.093593	−2.212075	−1.024026
C	0.196141	−3.418571	0.736850
C	−0.194207	−3.418216	−0.737438
C	−1.612295	0.079219	−0.111616
C	−1.534107	1.144461	0.819108
C	−2.876375	0.144057	−0.817892
C	1.612187	0.079678	0.111721
C	2.876383	0.144914	0.817865
C	3.669715	1.175917	0.447134
H	3.162136	−0.553294	1.589873
C	1.533597	1.144927	−0.818954
S	2.930921	2.138488	−0.803995
C	−3.670054	1.174905	−0.447411
S	−2.931848	2.137569	0.803959
H	−3.161888	−0.554238	−1.589905
H	−0.743417	1.334291	1.526042
H	−4.641748	1.443053	−0.829898
H	0.742983	1.334102	−1.526157
H	4.641354	1.444431	0.829502
H	2.121930	−2.453635	0.736329
H	0.724759	−4.333302	1.010189
H	−0.721753	−4.333365	−1.011447
H	−2.120861	−2.455163	−0.734589
H	1.129553	−2.023264	2.106010
H	−0.709087	−3.378299	1.349375
H	0.710926	−3.376372	−1.350009
H	−1.130594	−2.023658	−2.105375

C1–C1' = 3.38 Å

$E(S_1)$ = −1338.41596670 Ha

C	−0.662150	0.982112	0.278526
C	0.662205	0.982123	−0.278474
C	−1.135998	2.244564	0.975406
C	1.136075	2.244628	−0.975250
C	−0.227033	3.450924	0.727812

C	0.226874	3.450843	−0.727925
C	1.598557	−0.070328	−0.099418
C	1.483059	−1.153779	0.807692
C	2.873448	−0.143214	−0.783197
C	−1.598555	−0.070285	0.099350
C	−2.873303	−0.143336	0.783379
C	−3.641142	−1.196699	0.421742
H	−3.185455	0.565896	1.534865
C	−1.483152	−1.153631	−0.807893
S	−2.863099	−2.172257	−0.796564
C	3.641287	−1.196563	−0.421521
S	2.863028	−2.172369	0.796447
H	3.185714	0.566143	−1.534518
H	0.681270	−1.336917	1.503403
H	4.614097	−1.475389	−0.793260
H	−0.681544	−1.336552	−1.503872
H	−4.613851	−1.475642	0.793658
H	−2.148220	2.483728	0.633340
H	−0.766724	4.365755	0.978180
H	0.766383	4.365739	−0.978449
H	2.148175	2.483956	−0.632918
H	−1.228235	2.061556	2.054108
H	0.650803	3.410187	1.379120
H	−0.650937	3.409800	−1.379249
H	1.228638	2.061592	−2.053919

C1–C1' = 3.28 Å

$E(S_1)$ = −1338.41757474 Ha

C	−0.667533	1.008150	0.263406
C	0.667586	1.008132	−0.263417
C	−1.174292	2.275067	0.927059
C	1.174404	2.275031	−0.927058
C	−0.257058	3.481339	0.717657
C	0.257213	3.481332	−0.717670
C	1.584739	−0.061525	−0.088337
C	1.432710	−1.160915	0.795581
C	2.869402	−0.143457	−0.750288
C	−1.584731	−0.061474	0.088348
C	−2.869348	−0.143396	0.750390
C	−3.611842	−1.217775	0.397391
H	−3.207248	0.575060	1.481758
C	−1.432785	−1.160826	−0.795621
S	−2.795433	−2.203776	−0.788496
C	3.611876	−1.217828	−0.397225
S	2.795258	−2.203984	0.788386
H	3.207413	0.575056	−1.481548
H	0.621867	−1.335747	1.482395

H	4.585274	−1.507636	−0.758795
H	−0.621959	−1.335667	−1.482455
H	−4.585189	−1.507615	0.759074
H	−2.169282	2.510598	0.534603
H	−0.806860	4.396079	0.945268
H	0.807046	4.396055	−0.945272
H	2.169382	2.510540	−0.534556
H	−1.319971	2.097436	2.000425
H	0.592890	3.440724	1.405109
H	−0.592735	3.440749	−1.405125
H	1.320130	2.097384	−2.000414

C1–C1' = 3.18 Å

$E(S_1)$ = −1338.41911373 Ha

C	0.672530	1.033529	−0.247787
C	−0.672606	1.033407	0.248008
C	1.211163	2.304408	−0.876518
C	−1.211464	2.304193	0.876762
C	0.287383	3.510896	−0.705978
C	−0.288191	3.511019	0.705749
C	−1.570763	−0.052804	0.077051
C	−1.382297	−1.166626	−0.783367
C	−2.863830	−0.145352	0.718454
C	1.570749	−0.052663	−0.077120
C	2.863898	−0.144895	−0.718405
C	3.580989	−1.239603	−0.374860
H	3.226431	0.581085	−1.430351
C	1.382396	−1.166631	0.783136
S	2.727640	−2.233502	0.779515
C	−3.580672	−1.240251	0.375002
S	−2.727253	−2.233859	−0.779571
H	−3.226448	0.580501	1.430486
H	−0.566854	−1.330321	−1.466890
H	−4.553453	−1.541980	0.728315
H	0.566913	−1.330534	1.466569
H	4.553898	−1.541070	−0.728042
H	2.186364	2.535754	−0.434269
H	0.846813	4.425474	−0.909412
H	−0.848011	4.425429	0.908858
H	−2.186881	2.535129	0.434786
H	1.409800	2.132131	−1.941850
H	−0.532301	3.470849	−1.429414
H	0.531532	3.471598	1.429170
H	−1.409702	2.131974	1.942173

C1–C1' = 3.08 Å

$E(S_1) = -1338.42056415$ Ha

C	0.677185	1.058821	−0.231478
C	−0.677485	1.058531	0.231903
C	1.246697	2.333279	−0.823279
C	−1.247638	2.332802	0.823498
C	0.318130	3.540354	−0.692425
C	−0.319905	3.540468	0.692017
C	−1.556462	−0.043791	0.065070
C	−1.331034	−1.170408	−0.771811
C	−2.856423	−0.149186	0.687296
C	1.556557	−0.043206	−0.064881
C	2.856600	−0.147954	−0.687048
C	3.547084	−1.263272	−0.354621
H	3.243338	0.583665	−1.380320
C	1.331541	−1.170083	0.771764
S	2.657999	−2.262260	0.769599
C	−3.546474	−1.264721	0.354695
S	−2.657091	−2.263083	−0.769847
H	−3.243385	0.582109	1.380785
H	−0.514173	−1.318380	−1.456641
H	−4.517320	−1.579936	0.701411
H	0.514617	−1.318635	1.456386
H	4.518089	−1.578016	−0.701320
H	2.199270	2.560033	−0.331454
H	0.886772	4.454637	−0.869961
H	−0.889199	4.454432	0.869105
H	−2.200484	2.558767	0.331849
H	1.498436	2.166673	−1.877802
H	−0.468553	3.501462	−1.451800
H	0.466820	3.502520	1.451392
H	−1.498988	2.166371	1.878140

C1–C1' = 2.98 Å

$E(S_1) = -1338.42191001$ Ha

C	−0.681688	1.084251	0.214603
C	0.681972	1.084032	−0.214774
C	−1.281052	2.361653	0.767962
C	1.281769	2.361247	−0.768101
C	−0.349542	3.569638	0.677002
C	0.350779	3.569623	−0.676871
C	1.541890	−0.033726	−0.051656
C	1.278914	−1.171371	0.761876
C	2.846787	−0.154765	−0.656950
C	−1.541892	−0.033304	0.051683
C	−2.846755	−0.153996	0.657120
C	−3.508878	−1.290315	0.337064

H	−3.257361	0.580806	1.333183
C	−1.279309	−1.171009	−0.761901
S	−2.585057	−2.290339	−0.759525
C	3.508589	−1.291244	−0.336800
S	2.584234	−2.291202	0.759400
H	3.257684	0.579958	−1.332922
H	0.465796	−1.298940	1.454676
H	4.475725	−1.621728	−0.679551
H	−0.466370	−1.298693	−1.454893
H	−4.476041	−1.620576	0.679955
H	−2.208359	2.583495	0.227484
H	−0.926592	4.483543	0.827338
H	0.928233	4.483303	−0.827015
H	2.209240	2.582642	−0.227731
H	−1.585495	2.200777	1.808957
H	0.401911	3.532470	1.471444
H	−0.400701	3.532964	−1.471310
H	1.585997	2.200355	−1.809155

C1–C1' = 2.88 Å

$E(S_1)$ = −1338.42314215 Ha

C	0.686411	−1.109480	0.196284
C	−0.685991	−1.109785	−0.196167
C	1.316700	−2.389685	0.706057
C	−1.315674	−2.390236	−0.706109
C	0.384114	−3.598975	0.658637
C	−0.382622	−3.599168	−0.658578
C	−1.526817	0.023135	−0.037446
C	−1.226463	1.170432	0.752827
C	−2.834424	0.161752	−0.628574
C	1.526750	0.023803	0.037499
C	2.834403	0.162880	0.628501
C	3.467061	1.320182	0.322261
H	3.267928	−0.572960	1.289009
C	1.225913	1.170972	−0.752736
S	2.509658	2.318431	−0.748228
C	−3.467529	1.318839	−0.322526
S	−2.510624	2.317547	0.748277
H	−3.267636	−0.574224	−1.289152
H	−0.423650	1.274347	1.461028
H	−4.429539	1.665163	−0.663951
H	0.422842	1.274815	−1.460646
H	4.429024	1.666820	0.663513
H	2.213009	−2.606008	0.113173
H	0.969291	−4.512231	0.778611
H	−0.967461	−4.512649	−0.778492
H	−2.212003	−2.606909	−0.113397

H	1.678264	−2.235404	1.729107
H	−0.326922	−3.564155	1.489655
H	0.328409	−3.564150	−1.489591
H	−1.677111	−2.236078	−1.729229

C1–C1' = 2.78 Å

E(S_1) = −1338.42429826 Ha

C	−0.689606	1.133200	0.178337
C	0.689993	1.132907	−0.178462
C	−1.344266	2.415624	0.649181
C	1.345177	2.415045	−0.649374
C	−0.411444	3.625371	0.641097
C	0.413018	3.625316	−0.640904
C	1.512900	−0.013476	−0.023141
C	1.173766	−1.170099	0.742107
C	2.822901	−0.169632	−0.599757
C	−1.512916	−0.012910	0.023192
C	−2.823032	−0.168508	0.599699
C	−3.427518	−1.345221	0.308361
H	−3.279137	0.569247	1.242727
C	−1.174184	−1.169712	−0.741966
S	−2.438294	−2.342659	−0.735002
C	3.426920	−1.346592	−0.308450
S	2.437378	−2.343583	0.735037
H	3.279246	0.567910	−1.242859
H	0.386600	−1.248659	1.470760
H	4.383516	−1.708633	−0.648800
H	−0.386915	−1.248668	−1.470468
H	−4.384293	−1.706861	0.648632
H	−2.210634	2.627988	0.011762
H	−1.001594	4.538595	0.734140
H	1.003682	4.538233	−0.733696
H	2.211825	2.626880	−0.012167
H	−1.755709	2.266363	1.653569
H	0.262181	3.591667	1.502840
H	−0.260647	3.592247	−1.502638
H	1.756277	2.265687	−1.653891

C1–C1' = 2.68 Å

E(S_1) = −1338.42542188 Ha

C	−1.155956	−0.693199	0.158667
C	−1.155769	0.693403	−0.158804
C	−2.440205	−1.371989	0.587992
C	−2.439842	1.372553	−0.588088
C	−3.651211	−0.441579	0.620904

C	−3.651191	0.442566	−0.620698
C	0.002515	1.499940	−0.006246
C	1.169076	1.121792	0.730839
C	0.174978	2.812001	−0.568340
C	0.002150	−1.500000	0.006183
C	0.174178	−2.812245	0.567979
C	1.370929	−3.387150	0.295351
H	−0.565839	−3.292070	1.190945
C	1.168983	−1.121924	−0.730512
S	2.368422	−2.365363	−0.716717
C	1.371852	3.386635	−0.295696
S	2.368843	2.364903	0.716921
H	−0.564784	3.291831	−1.191607
H	1.224785	0.354846	1.483082
H	1.748042	4.337864	−0.635802
H	1.225083	−0.354742	−1.482484
H	1.746790	−4.338583	0.635255
H	−2.647762	−2.205075	−0.093955
H	−4.563939	−1.036430	0.684666
H	−4.563715	1.037751	−0.684258
H	−2.647009	2.205840	0.093722
H	−2.295805	−1.833448	1.570682
H	−3.619605	0.190692	1.513587
H	−3.620024	−0.189725	−1.513381
H	−2.295394	1.833779	−1.570884

C1–C1' = 2.58 Å

$E(S_1)$ = −1338.42658145 Ha

C	−1.178298	−0.696461	0.138066
C	−1.178231	0.696631	−0.138068
C	−2.463628	−1.396216	0.528365
C	−2.463508	1.396507	−0.528330
C	−3.675807	−0.469545	0.600170
C	−3.675750	0.469926	−0.600190
C	−0.009320	1.487699	0.013366
C	1.166906	1.069340	0.719439
C	0.179659	2.801633	−0.533618
C	−0.009471	−1.487658	−0.013370
C	0.179281	−2.801670	0.533502
C	1.396020	−3.345747	0.282560
H	−0.562903	−3.305841	1.134408
C	1.166871	−1.069409	−0.719321
S	2.393934	−2.290369	−0.694315
C	1.396487	3.345520	−0.282693
S	2.394169	2.290091	0.694368
H	−0.562412	3.305860	−1.134617
H	1.202513	0.324397	1.495281

H	1.786157	4.291322	−0.622870
H	1.202706	−0.324357	−1.495038
H	1.785500	−4.291665	0.622631
H	−2.667076	−2.196350	−0.193125
H	−4.588213	−1.067305	0.635788
H	−4.588113	1.067749	−0.635843
H	−2.666914	2.196608	0.193216
H	−2.323152	−1.902960	1.488752
H	−3.646041	0.121943	1.520501
H	−3.645985	−0.121560	−1.520523
H	−2.323001	1.903304	−1.488686

C1–C1' = 2.48 Å

$E(S_1)$ = −1338.42784300 Ha

C	−1.198706	−0.699045	0.118214
C	−1.198564	0.699345	−0.118023
C	−2.484963	−1.416160	0.472323
C	−2.484658	1.416695	−0.472259
C	−3.697775	−0.493757	0.580227
C	−3.697604	0.494490	−0.580346
C	−0.020575	1.476426	0.033035
C	1.164858	1.017931	0.705930
C	0.183733	2.792411	−0.497918
C	−0.020916	−1.476411	−0.033004
C	0.183095	−2.792476	0.497865
C	1.418218	−3.306822	0.269899
H	−0.561162	−3.320659	1.075238
C	1.164627	−1.018142	−0.705863
S	2.417246	−2.218763	−0.668773
C	1.418985	3.306475	−0.270012
S	2.417750	2.218267	0.668767
H	−0.560398	3.320718	−1.075339
H	1.183203	0.298109	1.506693
H	1.820475	4.247532	−0.609765
H	1.183186	−0.298214	−1.506520
H	1.819498	−4.247986	0.609605
H	−2.685376	−2.183922	−0.284293
H	−4.610141	−1.092625	0.590540
H	−4.609874	1.093502	−0.590807
H	−2.685045	2.184466	0.284356
H	−2.347938	−1.963701	1.410289
H	−3.668839	0.059484	1.524111
H	−3.668606	−0.058755	−1.524226
H	−2.347445	1.964244	−1.410196

C1–C1' = 2.38 Å

$E(S_1)$ = −1338.42920555 Ha

C	−1.216700	−0.701196	0.099163
C	−1.216618	0.701424	−0.099085
C	−2.503910	−1.433372	0.417506
C	−2.503728	1.433755	−0.417498
C	−3.717376	−0.516519	0.560212
C	−3.717321	0.517061	−0.560155
C	−0.030741	1.466005	0.051138
C	1.163357	0.968652	0.689383
C	0.187158	2.784012	−0.463685
C	−0.030942	−1.465953	−0.051136
C	0.186578	−2.784193	0.463259
C	1.437761	−3.271370	0.258194
H	−0.559549	−3.334996	1.016723
C	1.163368	−0.968666	−0.689029
S	2.438230	−2.152924	−0.641118
C	1.438433	3.270953	−0.258619
S	2.438481	2.152613	0.641301
H	−0.558746	3.334784	−1.017479
H	1.168424	0.276193	1.515350
H	1.849683	4.208120	−0.597597
H	1.168805	−0.275879	−1.514704
H	1.848714	−4.208783	0.596854
H	−2.701359	−2.167893	−0.372139
H	−4.629632	−1.115527	0.546191
H	−4.629498	1.116191	−0.546115
H	−2.701068	2.168364	0.372087
H	−2.370472	−2.020141	1.331708
H	−3.689169	−0.001161	1.525366
H	−3.689213	0.001693	−1.525306
H	−2.370213	2.020438	−1.331749

C1–C1' = 2.28 Å

$E(S_1)$ = −1338.43064454 Ha

C	−1.232631	−0.702750	0.081313
C	−1.232383	0.703188	−0.081006
C	−2.520780	−1.447611	0.364628
C	−2.520252	1.448456	−0.364520
C	−3.734967	−0.537306	0.539806
C	−3.734686	0.538531	−0.539954
C	−0.039463	1.456127	0.067763
C	1.162636	0.921098	0.669887
C	0.190350	2.776477	−0.430336
C	−0.039995	−1.456095	−0.067623
C	0.189218	−2.776778	0.429865
C	1.454152	−3.239633	0.247327

H	−0.558688	−3.348728	0.959064
C	1.162389	−0.921239	−0.669323
S	2.456463	−2.092957	−0.611685
C	1.455453	3.238904	−0.247888
S	2.457204	2.092233	0.611781
H	−0.557254	3.348475	−0.959910
H	1.158351	0.257193	1.520421
H	1.874470	4.173310	−0.585123
H	1.158466	−0.256932	−1.519555
H	1.872748	−4.174381	0.584142
H	−2.715001	−2.149135	−0.455239
H	−4.647061	−1.135573	0.501613
H	−4.646605	1.137076	−0.501957
H	−2.714420	2.150000	0.455345
H	−2.391311	−2.071131	1.254486
H	−3.708273	−0.059482	1.524154
H	−3.707921	0.060701	−1.524297
H	−2.390424	2.071979	−1.254322

C1–C1' = 2.18 Å

$E(S_1)$ = −1338.43212543 Ha

C	−1.246669	−0.704203	0.064637
C	−1.246736	0.704094	−0.064677
C	−2.535172	−1.459630	0.316112
C	−2.535284	1.459425	−0.316211
C	−3.750197	−0.556520	0.520875
C	−3.750291	0.556246	−0.520798
C	−0.047742	1.446616	0.082777
C	1.161549	0.875024	0.648031
C	0.192330	2.769432	−0.398052
C	−0.047593	−1.446613	−0.082764
C	0.192605	−2.769388	0.398133
C	1.469654	−3.209856	0.237676
H	−0.556550	−3.361135	0.903407
C	1.161651	−0.874939	−0.648035
S	2.473259	−2.037365	−0.581953
C	1.469366	3.209973	−0.237658
S	2.473077	2.037544	0.581939
H	−0.556883	3.361112	−0.903320
H	1.151126	0.240857	1.522547
H	1.894465	4.142480	−0.572727
H	1.151171	−0.240803	−1.522569
H	1.894832	−4.142324	0.572752
H	−2.726464	−2.130061	−0.530064
H	−4.662012	−1.153515	0.461149
H	−4.662124	1.153202	−0.460958
H	−2.726555	2.129982	0.529868

H	−2.408768	−2.115814	1.182362
H	−3.724638	−0.113210	1.521295
H	−3.724851	0.112924	−1.521216
H	−2.408942	2.115477	−1.182572

C1–C1' = 2.08 Å

$E(S_1)$ = −1338.43367325 Ha

C	−1.259707	−0.704849	0.048803
C	−1.259303	0.705136	−0.048622
C	−2.548659	−1.468369	0.270332
C	−2.547832	1.469310	−0.270350
C	−3.764130	−0.572360	0.502362
C	−3.763811	0.573959	−0.502330
C	−0.054491	1.437609	0.097515
C	1.161401	0.829860	0.624558
C	0.194901	2.762624	−0.365849
C	−0.055281	−1.437812	−0.097420
C	0.193736	−2.762920	0.365829
C	1.481336	−3.183838	0.228388
H	−0.557395	−3.372961	0.846042
C	1.160703	−0.830370	−0.624548
S	2.487559	−1.987572	−0.550017
C	1.482662	3.183159	−0.228753
S	2.488535	1.986885	0.550093
H	−0.556136	3.372863	−0.845956
H	1.148536	0.227215	1.522820
H	1.912550	4.114112	−0.562139
H	1.147959	−0.227720	−1.522811
H	1.910994	−4.114972	0.561561
H	−2.737332	−2.109087	−0.599150
H	−4.675912	−1.167025	0.422031
H	−4.675258	1.169136	−0.421995
H	−2.736176	2.110308	0.598992
H	−2.425124	−2.153982	1.113752
H	−3.739802	−0.162023	1.516802
H	−3.739743	0.163589	−1.516763
H	−2.423835	2.154688	−1.113894

C1–C1' = 1.98 Å

$E(S_1)$ = −1338.43550870 Ha

C	1.271284	0.705570	0.033395
C	1.271418	−0.705394	−0.033478
C	2.559893	1.476498	0.226519
C	2.560143	−1.476190	−0.226573
C	3.776536	0.588937	0.484070

C	3.776606	−0.588411	−0.484204
C	0.061651	−1.429252	0.111309
C	−1.160865	−0.786380	0.599325
C	−0.194765	−2.756738	−0.332598
C	0.061466	1.429338	−0.111379
C	−0.195340	2.756530	0.333269
C	−1.492531	3.159856	0.220192
H	0.557918	3.384210	0.786977
C	−1.160894	0.786335	−0.599703
S	−2.502558	1.941519	−0.515489
C	−1.491918	−3.160246	−0.219382
S	−2.502396	−1.941750	0.515105
H	0.558672	−3.384557	−0.785803
H	−1.148207	−0.217826	1.521384
H	−1.924082	−4.091245	−0.549783
H	−1.148021	0.218007	−1.521900
H	−1.925010	4.090517	0.551114
H	2.745479	2.088956	−0.663805
H	4.687788	1.181445	0.383900
H	4.687981	−1.180743	−0.384111
H	2.745933	−2.088574	0.663769
H	2.438257	2.188784	1.047719
H	3.753933	0.210535	1.510889
H	3.753845	−0.210006	−1.511021
H	2.438675	−2.188525	−1.047760

C1–C1' = 1.88 Å

$E(S_1)$ = −1338.43897957 Ha

C	1.281665	0.706240	0.021156
C	1.281656	−0.706311	−0.021067
C	2.570341	1.481548	0.194010
C	2.570317	−1.481629	−0.193982
C	3.787073	0.599636	0.470552
C	3.787047	−0.599726	−0.470569
C	0.068423	−1.421648	0.124463
C	−1.162008	−0.743459	0.573384
C	−0.194837	−2.753468	−0.297727
C	0.068448	1.421601	−0.124385
C	−0.194704	2.753526	0.297530
C	−1.497838	3.142068	0.208418
H	0.562033	3.398620	0.720364
C	−1.162026	0.743434	−0.573227
S	−2.516529	1.899328	−0.478801
C	−1.497989	−3.141945	−0.208629
S	−2.516575	−1.899271	0.478866
H	0.561839	−3.398522	−0.720733
H	−1.150802	−0.211495	1.518908

H	−1.931695	−4.075463	−0.529909
H	−1.150851	0.211441	−1.518737
H	−1.931472	4.075669	0.529553
H	2.754630	2.072086	−0.711213
H	4.698407	1.189508	0.356297
H	4.698380	−1.189607	−0.356353
H	2.754640	−2.072188	0.711223
H	2.450493	2.213141	0.998180
H	3.765152	0.244774	1.505760
H	3.765085	−0.244859	−1.505775
H	2.450426	−2.213208	−0.998159

C1–C1' = 1.78 Å

$E(S_1)$ = −1338.44171502 Ha

C	1.291819	0.706223	0.009321
C	1.291818	−0.706223	−0.009384
C	2.580177	1.485928	0.158311
C	2.580185	−1.485926	−0.158311
C	3.797781	0.611671	0.454957
C	3.797801	−0.611670	−0.454903
C	0.074540	−1.414452	0.133827
C	−1.162461	−0.703181	0.543643
C	−0.192294	−2.751243	−0.259448
C	0.074539	1.414453	−0.133872
C	−0.192283	2.751246	0.259400
C	−1.502097	3.127488	0.194166
H	0.568056	3.414240	0.646969
C	−1.162477	0.703179	−0.543642
S	−2.532088	1.863301	−0.432962
C	−1.502106	−3.127485	−0.194177
S	−2.532075	−1.863305	0.433000
H	0.568034	−3.414235	−0.647043
H	−1.156419	−0.214188	1.514056
H	−1.933186	−4.066452	−0.503275
H	−1.156462	0.214179	−1.514053
H	−1.933166	4.066460	0.503261
H	2.761651	2.052922	−0.762490
H	4.708679	1.198794	0.324137
H	4.708692	−1.198794	−0.324039
H	2.761618	−2.052921	0.762499
H	2.462066	2.238048	0.943544
H	3.777603	0.283637	1.499005
H	3.777674	−0.283638	−1.498953
H	2.462108	−2.238047	−0.943548

C1–C1' = 1.68 Å

$E(S_1)$ = −1338.44435462 Ha

C	1.301173	0.706011	0.000775
C	1.301169	−0.706018	−0.000706
C	2.589079	1.488982	0.127575
C	2.589066	−1.488994	−0.127565
C	3.807624	0.621824	0.440994
C	3.807598	−0.621843	−0.441051
C	0.080303	−1.408040	0.138070
C	−1.162745	−0.666009	0.509968
C	−0.187901	−2.752053	−0.215388
C	0.080310	1.408034	−0.138009
C	−0.187894	2.752059	0.215410
C	−1.503651	3.118692	0.172937
H	0.576687	3.434303	0.559114
C	−1.162728	0.666010	−0.509950
S	−2.548358	1.831847	−0.376631
C	−1.503664	−3.118671	−0.172976
S	−2.548377	−1.831841	0.376612
H	0.576684	−3.434295	−0.559090
H	−1.164239	−0.226877	1.505933
H	−1.929556	−4.067122	−0.460125
H	−1.164191	0.226881	−1.505915
H	−1.929543	4.067160	0.460028
H	2.767379	2.034665	−0.806765
H	4.718072	1.206168	0.295359
H	4.718053	−1.206190	−0.295468
H	2.767408	−2.034667	0.806771
H	2.472981	2.259039	0.895473
H	3.789378	0.317515	1.492232
H	3.789292	−0.317534	−1.492288
H	2.472928	−2.259058	−0.895451

C1–C1' = 1.58 Å

$E(S_1)$ = −1338.44547567 Ha

C	1.309770	0.705752	−0.004191
C	1.309768	−0.705755	0.004211
C	2.597048	1.490984	0.104100
C	2.597042	−1.490991	−0.104098
C	3.816601	0.629630	0.429869
C	3.816598	−0.629641	−0.429873
C	0.085787	−1.402285	0.136358
C	−1.161950	−0.631899	0.472191
C	−0.183101	−2.754992	−0.164763
C	0.085790	1.402281	−0.136338
C	−0.183090	2.755001	0.164734
C	−1.504405	3.113497	0.143099

H	0.585356	3.457736	0.454768
C	−1.161947	0.631895	−0.472163
S	−2.564243	1.802591	−0.308979
C	−1.504417	−3.113485	−0.143136
S	−2.564248	−1.802588	0.308988
H	0.585341	−3.457719	−0.454826
H	−1.173221	−0.248909	1.492996
H	−1.924443	−4.074184	−0.397079
H	−1.173214	0.248905	−1.492968
H	−1.924425	4.074213	0.396987
H	2.772153	2.019939	−0.840556
H	4.726566	1.211632	0.272252
H	4.726560	−1.211646	−0.272256
H	2.772154	−2.019955	0.840551
H	2.482706	2.274653	0.858340
H	3.800340	0.344213	1.486421
H	3.800335	−0.344225	−1.486425
H	2.482689	−2.274652	−0.858344

References

(1) Casida, M. E.; Huix-Rotllant, M. Progress in Time-Dependent Density-Functional Theory. *Annu. Rev. Phys. Chem.* **2012**, *63*, 287–323.

(2) Gaussian 16, Revision B.01, Frisch, M. J.; Trucks, G. W.; Schlegel, H. B.; Scuseria, G. E.; Robb, M. A.; Cheeseman, J. R.; Scalmani, G.; Barone, V.; Petersson, G. A.; Nakatsuji, H.; Li, X.; Caricato, M.; Marenich, A. V.; Bloino, J.; Janesko, B. G.; Gomperts, R.; Mennucci, B.; Hratchian, H. P.; Ortiz, J. V.; Izmaylov, A. F.; Sonnenberg, J. L.; Williams-Young, D.; Ding, F.; Lipparini, F.; Egidi, F.; Goings, J.; Peng, B.; Petrone, A.; Henderson, T.; Ranasinghe, D.; Zakrzewski, V. G.; Gao, J.; Rega, N.; Zheng, G.; Liang, W.; Hada, M.; Ehara, M.; Toyota, K.; Fukuda, R.; Hasegawa, J.; Ishida, M.; Nakajima, T.; Honda, Y.; Kitao, O.; Nakai, H.; Vreven, T.; Throssell, K.; Montgomery, J. A., Jr.; Peralta, J. E.; Ogliaro, F.; Bearpark, M. J.; Heyd, J. J.; Brothers, E. N.; Kudin, K. N.; Staroverov, V. N.; Keith, T. A.; Kobayashi, R.; Normand, J.; Raghavachari, K.; Rendell, A. P.; Burant, J. C.; Iyengar, S. S.; Tomasi, J.; Cossi, M.; Millam, J. M.; Klene, M.; Adamo, C.; Cammi, R.; Ochterski, J. W.; Martin, R. L.; Morokuma, K.; Farkas, O.; Foresman, J. B.; Fox, D. J. Gaussian, Inc., Wallingford CT, **2016**.

(3) Oruganti, B.; Kalapos, P. P.; Bhargav, V.; London, G.; Durbeej, B. Photoinduced Changes in Aromaticity Facilitate Electrocyclization of Dithienylbenzene Switches. *J. Am. Chem. Soc.* **2020**, *142*, 13941–13953.

(4) Irie, M. Diarylethenes for Memories and Switches. *Chem. Rev.* **2000**, *100*, 1685–1716.

(5) Marenich, A. V.; Cramer, C. J.; Truhlar, D. G. Universal Solvation Model Based on Solute Electron Density and on a Continuum Model of the Solvent Defined by the Bulk Dielectric Constant and Atomic Surface Tensions. *J. Phys. Chem. B* **2009**, *113*, 6378–6396.

(6) (a) Andersson, K.; Malmqvist, P.-Å.; Roos, B. O. Second-Order Perturbation Theory with a Complete Active Space Self-Consistent Field Reference Function. *J. Chem. Phys.* **1992**, *96*, 1218–1226. (b) Finley, J.; Malmqvist, P.-Å.; Roos, B. O.; Serrano-Andrés, L. The Multi-State CASPT2 Method. *Chem. Phys. Lett.* **1998**, *288*, 299–306.

(7) (a) Hirata, S.; Head-Gordon, M. Time-Dependent Density Functional Theory within the Tamm-Dancoff Approximation. *Chem. Phys. Lett.* **1999**, *314*, 291–299. (b) Chantzis, A.; Laurent, A. D.; Adamo, C.; Jacquemin, D. Is the Tamm-Dancoff Approximation Reliable for the Calculation of Absorption and Fluorescence Band Shapes? *J. Chem. Theory Comput.* **2013**, *9*, 4517–4525.

(8) Zhao, Y.; Truhlar, D. G. The M06 Suite of Density Functionals for Main Group

Thermochemistry, Thermochemical Kinetics, Noncovalent Interactions, Excited States, and Transition Elements: Two New Functionals and Systematic Testing of Four M06-Class Functionals and 12 Other Functionals. *Theor. Chem. Acc.* **2008**, *120*, 215–241.

(9) Chai, J.-D.; Head-Gordon, M. Long-Range Corrected Hybrid Density Functionals with Damped Atom-Atom Dispersion Corrections. *Phys. Chem. Chem. Phys.* **2008**, *10*, 6615–6620.

(10) Roos, B. O.; Taylor, P. R.; Siegbahn, P. E. M. A Complete Active Space SCF Method (CASSCF) Using a Density Matrix Formulated Super-CI Approach. *Chem. Phys.* **1980**, *48*, 157–173.

(11) Scalmani, G.; Frisch, M. J.; Mennucci, B.; Tomasi, J.; Cammi, R.; Barone, V. Geometries and Properties of Excited States in the Gas Phase and in Solution: Theory and Application of a Time-Dependent Density Functional Theory Polarizable Continuum Model. *J. Chem. Phys.* **2006**, *124*, 094107.

(12) (a) Liu, J.; Liang, W. Analytical Approach for the Excited-State Hessian in Time-Dependent Density Functional Theory: Formalism, Implementation, and Performance. *J. Chem. Phys.* **2011**, *135*, 184111. (b) Liu, J.; Liang, W. Analytical Second Derivatives of Excited-State Energy within the Time-Dependent Density Functional Theory Coupled with a Conductor-Like Polarizable Continuum Model. *J. Chem. Phys.* **2013**, *138*, 024101.

(13) (a) Barbatti, M. Nonadiabatic Dynamics with Trajectory Surface Hopping Method. *WIREs Comput. Mol. Sci.* **2011**, *1*, 620–633. (b) Tapavicza, E.; Bellchambers, G. D.; Vincent, J. C.; Furche, F. Ab Initio Non-Adiabatic Molecular Dynamics. *Phys. Chem. Chem. Phys.* **2013**, *15*, 18336–18348. (c) Curchod, B. F. E.; Martínez, T. J. Ab Initio Nonadiabatic Quantum Molecular Dynamics. *Chem. Rev.* **2018**, *118*, 3305–3336.

(14) Tully, J. C. Molecular Dynamics with Electronic Transitions. *J. Chem. Phys.* **1990**, *93*, 1061–1071.

(15) (a) Tapavicza, E.; Meyer, A. M.; Furche, F. Unravelling the Details of Vitamin D Photosynthesis by Non-Adiabatic Molecular Dynamics Simulations. *Phys. Chem. Chem. Phys.* **2011**, *13*, 20986–20998. (b) Furche, F.; Ahlrichs, R.; Hättig, C.; Klopper, W.; Sierka, M.; Weigend, F. Turbomole. *WIREs Comput. Mol. Sci.* **2014**, *4*, 91–100.

(16) Verlet, L. Computer "Experiments" on Classical Fluids. I. Thermodynamical Properties of Lennard-Jones Molecules. *Phys. Rev.* **1967**, *159*, 98–103.

(17) Cordova, F.; Doriol, L. J.; Ipatov, A.; Casida, M. E.; Filippi, C.; Vela, A. Troubleshooting Time-Dependent Density-Functional Theory for Photochemical Applications: Oxirane. *J. Chem. Phys.* **2007**, *127*, 164111.

(18) (a) Schleyer, P. v. R.; Maerker, C.; Dransfeld, A.; Jiao, H.; van Eikema Hommes, N. J. R.

Nucleus-Independent Chemical Shifts : A Simple and Efficient Aromaticity Probe. *J. Am. Chem. Soc.* **1996**, *118*, 6317–6318. (b) Fallah-Bagher-Shaidaei, H.; Wannere, C. S.; Corminboeuf, C.; Puchta, R.; Schleyer, P. v. R. Which NICS Aromaticity Index for Planar π Rings Is Best? *Org. Lett.* **2006**, *8*, 863–866.

(19) (a) Stanger, A. Nucleus-Independent Chemical Shifts (NICS): Distance Dependence and Revised Criteria for Aromaticity and Antiaromaticity. *J. Org. Chem.* **2006**, *71*, 883–893. (b) Gershoni-Poranne, R.; Stanger, A. The NICS-XY-Scan: Identification of Local and Global Ring Currents in Multi-Ring Systems. *Chem. Eur. J.* **2014**, *20*, 5673–5688. (c) Yuan, B.; Zhuang, J.; Kirmess, K. M.; Bridgmohan, C. N.; Whalley, A. C.; Wang, L.; Plunkett, K. N. Pentaleno[1,2-*a*:4,5']diacenaphthylenes: Uniquely Stabilized Pentalene Derivatives. *J. Org. Chem.* **2016**, *81*, 8312–8318. (d) Stanger, A. Reexamination of $NICS_{\pi,zz}$: Height Dependence, Off-Center Values, and Integration. *J. Phys. Chem. A* **2019**, *123*, 3922–3927. (e) Stanger, A. NICS – Past and Present. *Eur. J. Org. Chem.* **2020**, 3120–3127.

(20) Noorizadeh, S.; Shakerzadeh, E. Shannon Entropy as a New Measure of Aromaticity, Shannon Aromaticity. *Phys. Chem. Chem. Phys.* **2010**, *12*, 4742–4749.

(21) Bader, R. F. W. A Quantum Theory of Molecular Structure and Its Applications. *Chem. Rev.* **1991**, *91*, 893–928.

(22) Shannon, C. E. A Mathematical Theory of Communication. *Bell Syst. Tech. J.* **1948**, *27*, 379–423.

(23) (a) Krygowski, T. M. Crystallographic Studies of Inter- and Intramolecular Interactions Reflected in Aromatic Character of π-Electron Systems. *J. Chem. Inf. Comput. Sci.* **1993**, *33*, 70–78. (b) Krygowski, T. M.; Cyrański, M. K. Structrual Aspects of Aromaticity. *Chem. Rev.* **2001**, *101*, 1385–1419.

(24) Fdez. Galván, I.; Vacher, M.; Alavi, A.; Angeli, C.; Aquilante, F.; Autschbach, J.; Bao, J. J.; Bokarev, S. I.; Bogdanov, N. A.; Carlson, R. K.; Chibotaru, L. F.; Creutzberg, J.; Dattani, N.; Delcey, M. G.; Dong, S. S.; Dreuw, A.; Freitag, L.; Frutos, L. M.; Gagliardi, L.; Gendron, F.; Giussani, A.; González, L.; Grell, G.; Guo, M.; Hoyer, C. E.; Johansson, M.; Keller, S.; Knecht, S.; Kovačević, G.; Källman, E.; Manni, G. L.; Lundberg, M.; Ma, Y.; Mai, S.; Malhado, J. P.; Malmqvist, P. Å.; Marquetand, P.; Mewes, S. A.; Norell, J.; Olivucci, M.; Oppel, M.; Phung, Q. M.; Pierloot, K.; Plasser, F.; Reiher, M.; Sand, A. M.; Schapiro, I.; Sharma, P.; Stein, C. J.; Sørensen, L. K.; Truhlar, D. G.; Ugandi, M.; Ungur, L.; Valentini, A.; Vancoillie, S.; Veryazov, V.; Weser, O.; Wesołowski, T. A.; Widmark, P.-O.; Wouters, S.; Zech, A.; Zobel, J. P.; Lindh, R. OpenMolcas: From Source Code to Insight. *J. Chem. Theory Comput.* **2019**, *15*, 5925–5964.

(25) TURBOMOLE V7.4 2019, a Development of University of Karlsruhe and Forschungszentrum Karlsruhe GmbH, 1989-2007, TURBOMOLE GmbH, since 2007; available from http://www.turbomole.com (accessed April 25, 2022).

(26) (a) Aidas, K.; Angeli, C.; Bak, K. L.; Bakken, V.; Bast, R.; Boman, L.; Christiansen, O.; Cimiraglia, R.; Coriani, S.; Dahle, P.; Dalskov, E. K.; Ekström, U.; Enevoldsen, T.; Eriksen, J. J.; Ettenhuber, P.; Fernández, B.; Ferrighi, L.; Fliegl, H.; Frediani, L.; Hald, K. ; Halkier, A.; Hättig, C.; Heiberg, H.; Helgaker, T.; Hennum, A. C.; Hettema, H.; Hjertenæs, E.; Høst, S.; Høyvik, I.-M.; Iozzi, M. F.; Jansik, B.; Jensen, H. J. A.; Jonsson, D.; Jørgensen, P.; Kauczor, J.; Kirpekar, S.; Kjærgaard, T.; Klopper, W.; Knecht, S.; Kobayashi, R.; Koch, H.; Kongsted, J.; Krapp, A.; Kristensen, K.; Ligabue, A.; Lutnæs, O. B.; Melo, J. I.; Mikkelsen, K. V.; Myhre, R. H.; Neiss, C.; Nielsen, C. B.; Norman, P.; Olsen, J.; Olsen, J. M. H.; Osted, A.; Packer, M. J.; Pawlowski, F.; Pedersen, T. B.; Provasi, P. F.; Reine, S.; Rinkevicius, Z.; Ruden, T. A.; Ruud, K.; Rybkin, V. V.; Salek, P.; Samson, C. C. M.; de Merás, A. S.; Saue, T.; Sauer, S. P. A.; Schimmelpfennig, B.; Sneskov, K.; Steindal, A. H.; Sylvester-Hvid, K. O.; Taylor, P. R.; Teale, A. M.; Tellgren, E. I.; Tew, D. P.; Thorvaldsen, A. J.; Thøgersen, L.; Vahtras, O.; Watson, M. A.; Wilson, D. J. D.; Ziolkowski, M.; Ågren, H. The Dalton Quantum Chemistry Program System. *WIREs Comput. Mol. Sci.* **2014**, *4*, 269–284. (b) Dalton, A Molecular Electronic Structure Program, Release Dalton 2016.2 (2016), see http://daltonprogram.org (accessed July 25, 2022).

(27) Lu, T.; Chen, F. Multiwfn: A Multifunctional Wavefunction Analyzer. *J. Comput. Chem.* **2012**, *33*, 580–592.
